# Functional Analysis of the Chemosensory Protein GmolCSP8 From the Oriental Fruit Moth, *Grapholita molesta* (Busck) (Lepidoptera: Tortricidae)

**DOI:** 10.3389/fphys.2019.00552

**Published:** 2019-05-07

**Authors:** Guang-Wei Li, Xiu-Lin Chen, Li-Hui Chen, Wen-Qiang Wang, Jun-Xiang Wu

**Affiliations:** ^1^Shaanxi Province Key Laboratory of Jujube, Yan’an University, Yan’an, China; ^2^College of Life Sciences, Yan’an University, Yan’an, China; ^3^Key Laboratory of Plant Protection Resources and Pest Management (Northwest A&F University), Ministry of Education, Yangling, China

**Keywords:** *Grapholita molesta*, chemosensory protein, chemoreception, fluorescence ligand-binding assays, molecular docking, site-directed mutagenesis

## Abstract

Chemosensory proteins (CSPs) belong to a family of small water-soluble proteins that can selectively bind and transport odorant molecules for olfactory communication in insects. To date, their definite physiological functions in olfaction remain controversial when compared with odorant binding proteins (OBPs). To investigate the functions of CSPs in the oriental fruit moth *Grapholita molesta*, we determined the tissue expression patterns and binding properties of the CSP, GmolCSP8. The key binding sites of GmolCSP8 with a representative ligand were evaluated using molecular flexible docking, site-directed mutagenesis and ligand-binding experiments. Multiple sequence alignment and phylogenetic analysis showed that GmolCSP8 possesses a typical conserved four cysteines motif and shares high sequence identity with some CSP members of other Lepidopteran insects. GmolCSP8 was predominantly expressed in the wings and antennae of both male and female adults and may be involve in contact chemoreception. Recombinant GmolCSP8 (rGmolCSP8) exhibited specific-binding affinities to small aliphatic alcohols (C4–12) and had the strongest binding affinity to 1-hexanol. The three-dimensional structure of GmolCSP8 was constructed using the structure of sgCSP4 as a template. Site-directed mutagenesis and ligand-binding experiments confirmed that Thr27 is the key binding site in GmolCSP8 for 1-hexanol binding, because this residue can form hydrogen bond with the oxygen atom of the hydroxyl group in 1-hexanol, and Leu30 may play an important role in binding to 1-hexanol. We found that pH significantly affected the binding affinities of rGmolCSP8 to ligand, revealing that ligand-binding and -release by this protein is related to a pH-dependent conformational transition. Based on these results, we infer that GmolCSP8 may participate in the recognition and transportation of 1-hexanol and other small aliphatic alcohols.

## Introduction

The oriental fruit moth, *Grapholita molesta* Busck (Lepidoptera: Tortricidae), is a major pest species of fruit trees belonging to the family Rosacea, and has caused substantial losses in fruit yields throughout the world (Myers et al., 2007). The adult moths exhibit pronounced seasonal host-transfer behavior in adjacent areas of peach and pear, or peach and apple orchards (Rajapakse et al., 2006). The larvae mainly infest peach shoots in the early growing season and then shift to damage pear or apple fruits in the vicinity of peach orchards in the mid-to-late season (Piñero and Dorn, 2009; Lu et al., 2012; Najar-Rodriguez et al., 2013a). Selection of host location and oviposition behaviors of *G. molesta* are mediated by semiochemicals [e.g., (*Z*)-3-hexen-1-ol, 1-hexanol, (*E*)-2-hexenal, hexanal, heptanal, octanal, nonanal, benzaldehyde, (*Z*)-3-hexen-1-yl acetate, butyl hexanoate, (*Z*)-β-ocimene, and (*E*)-β-farnesene] emitted from host-plants (Natale et al., 2003; Il’ichev et al., 2009; Piñero et al., 2008). Changes in the components of host-plant volatiles are the primary cause of host transfer for this pest (Najar-Rodriguez et al., 2013b). *G. molesta* is predominantly monitored and controlled by sex pheromone trapping of male moths. However, this species is able to undergo multiple mating (up to fours), and the females can present a higher mating rate in orchards controlled by sex attractants (de Morais et al., 2012). In addition, the females, especially gravid females exhibit stronger flight performance than males, and have the capacity for inter-orchard flights (Steiner and Yetter, 1933). Female *G. molesta* are considered the main colonists and can cause a serious threat to pear and apple orchards in the vicinity of peach crops. Development of a control strategy for female *G. molesta* based on host-plant-volatiles is very promising. Thus, investigating the function of olfaction-related genes will help to better understand the olfactory system of *G. molesta*.

The insect olfactory system is highly specific and sensitive, and can detect and discriminate semiochemicals from complex environments and transfer signals to the nervous system to initiate specific behavioral responses, such as identifying conspecifics, finding mates, mating with partners, locating host plants, selecting oviposition sites, and avoiding toxins and predators (Sato and Touhara, 2009; Antony et al., 2016). Olfaction perception in insects is regulated through numerous olfactory proteins present in the sensilla lymph of the antennae, including chemosensory proteins (CSPs), odorant binding proteins (OBPs), odorant receptors (ORs), ionotropic receptors (IRs), sensory neuron membrane proteins (SNMPs), and odorant degrading enzymes (ODEs) (Bruyne and Baker, 2008). CSPs and OBPs are small-soluble carrier proteins, which function during the initial stages of chemoreception in insects. Members of these two groups of proteins can selectively bind and transport hydrophobic odorant molecules across the aqueous sensillum lymph to ORs located on the dendrite membranes of olfactory receptor neuron (ORN) cells, activating olfactory-related signal transduction (Laughlin et al., 2008).

Compared with the expression patterns of OBPs, which are specific or highly expressed in antennae, CSPs are expressed in chemosensory tissues, including antennae (Jacquin-Joly et al., 2001; Liu et al., 2010; Zhang et al., 2013, 2014), labial palps (Jin et al., 2005), maxillary palps (Maleszka and Stange, 1997), proboscis (Nagnan-Le et al., 2000), legs (Wanner et al., 2004), and wings (Zhou et al., 2008), and in non-chemosensory tissues, such as the ejaculatory bulb (Dyanov and Dzitoeva, 1995; Sirot et al., 2008), and pheromone gland (Jacquin-Joly et al., 2001) in numerous insect species, suggesting multiple roles, beyond chemosensation. Abundant CSPs in antennae are involved in the perception and recognition of sex pheromones or host-plant volatiles. For example, *Monochamus alternatus* MaltCSP5 exhibited strong binding affinities to pine volatile components myrcene, (+)-β-pinene, and (-)isolongifolene (Ali et al., 2019); *Mythimna separata* MsepCSP8 can bind to plant volatiles hexanal and α-terpinene (Younas et al., 2018); and *Microplitis mediator* MmedCSP3 showed high binding affinities to sex pheromone components *cis*-11-hexadecenyl aldehyde (*Z*11-16: Ald), *cis*-11-hexadecanol (*Z*11-16: OH), and *trans*-11-tetradecenyl acetate (*E*11-14: Ac) (Peng et al., 2017). RNAi and behavioral tests showed that *Nilaparvata lugens* NlugCSP8 is involved in the perception of plant volatiles nerolidol, hexanal, and *trans-*2-hexenal (Waris et al., 2018). The non-olfactory functions of insect CSPs have also been documented, and include embryonic integument formation in *Apis mellifera* (Maleszka et al., 2007), leg regeneration in *Periplaneta americana* (Kitabayashi et al., 1998), tissue-remodeling in *Drosophila melanogaster* (Sabatier et al., 2003), and regulation of circadian cycles in *Frankliniella occidentalis* (Claridge-Chang et al., 2001). The proposed olfactory function of insect CSPs is supported by genes expression in the lymph of chemosensilla and ligand-binding properties of recombinant proteins to odor molecules observed *in vitro*, however, owing to a lack of direct evidence on the molecular recognition of semiochemicals by CSPs, their exact physiological functions and molecular mechanisms still remains unclear.

In our previous study, we identified 17 CSP genes from the antennae of *G. molesta* using RNA-Sequencing (RNA-seq); five of these were selected with reads per kilobase per million mapped reads (RPM) value that was high in the antennae transcriptome, and successfully expressed in a prokaryotic expression system. Ligand-binding assays demonstrated that four recombinant GmolCSPs (rGmolCSPs) exhibited only weak binding affinities to host plant volatiles (Li G.W. et al., 2015). In this study, we found that a CSP, GmolCSP8, demonstrated binding specificity to small aliphatic alcohols. The main objective of this study was to identify the functions of GmolCSP8 during chemoreception. We performed real-time quantitative PCR (qRT-PCR) to monitor the expression of GmolCSP8 in different tissues of the adult males and females. Binding properties of GmolCSP8 were assessed using four sex pheromones and host-plant volatiles as putative ligands. Based on the results of ligand-binding assays, we combined homology modeling, molecular docking, site-directed mutagenesis, and ligand-binding assays to investigate the binding sites of GmolCSP8 to 1-hexanol and proposed a possible ligand-binding mechanism.

## Materials and Methods

### Insect Rearing and Tissue Collection

*Grapholita molesta* in all experiments were obtained from our laboratory colony, which has been maintained for more than 90 generations at the College of Plant Protection, Northwest A&F University, Yangling, China. The larvae were reared on an artificial diet and kept in an artificial climate chest under conditions of 25 ± 1°C, 70 ± 5% relative humidity (RH), and a 15:9 (light: dark) photoperiod (Du et al., 2010). Pupae were sexed, and male and female moths were kept in separate glass jar after emergence. The adults were fed a 5% honey solution and maintained under the conditions described above. To determine the tissue expression profiles of the target CSP gene in *G. molesta*, the antennae (200 moths per sample), heads (without antennae; 40 moths per sample), thoraxes (without wings and legs; 10 moths each sample), abdomens (five moths per sample), legs (100 moths per sample), and wings (100 moths per sample) from both sexes were excised (three replicates) from 3 to 4-day-old moths and immediately transferred to 1.5 mL centrifuge tubes immersed in liquid nitrogen and prepared for total RNA isolation.

### RNA Extraction and cDNA Synthesis

Total RNA from each sample was isolated using RNAiso Plus extraction reagent (TaKaRa, Shiga, Japan). Total RNA integrity was evaluated using 1.5% agarose electrophoresis, and the concentration was quantified by a spectrophotometry (SimpliNano, GE, United States). Residual genomic DNA was removed using DNase I (Thermo Scientific, United States), and 1 μg of RNA was reverse-transcribed into first-strand cDNA with an oligo (dT)18 primer using Revertaid First Strand cDNA Synthesis Kit (Thermo Fisher Scientific, Waltham, MA, United States). The products were stored at -80°C.

### ORF Amplification and Phylogenetic Analysis

Based on the annotated Unigene from the antennal transcriptome of *G. molesta* (Li G.W. et al., 2015), full-length cDNA encoding GmolCSP8 was amplified with female antennae cDNA using ExTaq DNA polymerase (Thermo Scientific, United States) and a pair of specific-primers (Table 1). PCR conditions were as follows: initial denaturation at 95°C for 5 min, followed by 35 cycles of 95°C for 30 s, 58°C for 30 s, and 72°C for 30 s, and final extension at 72°C for 10 min. The PCR products were gel-purified with DNA Purification Kit (TianGen, Beijing, China), and ligated into a pMD^®^19-T vector (TaKaRa, Dalian, China), and then were transformed into DH5α *Escherichia coli* competent cells (TianGen, Beijing, China). These were then sequenced (Aoke Biotech Company, Xi’an, China) to obtain the correct sequence. Sequence similarities between GmolCSP8 and various homologs from different insects were calculated via NCBI BLASTX programs. Amino acid sequences were aligned by DNAMAN 6.0 software (Lynnon Biosoft, San Ramon, CA, United States). N-terminal signal peptides were predicted using the online SignalP 4.0 server^1^ (Petersen et al., 2011). The isoelectric point (pI) and molecular weight (Mw) of GmolCSP8 were calculated with the ExPASy server program “Compute pI/Mw”^2^ (Wilkins et al., 1999). Based on sequence alignment obtained via MAFFT 6.0 software (CBRC, Misawa, Miyata, Japan) (Kuraku et al., 2013), a phylogenetic tree was constructed by Seaview 4.0 software (Prabi, Paris, French) (Gouy et al., 2010) using the neighbor-joining method with a p-distance model and pairwise gap deletion. Bootstrapping was performed to estimate the reliability of branches using 1,000 replicates. GenBank accession numbers of 104 CSPs and corresponding amino acid sequences used in the phylogenetic analyses are listed in Supplementary Data Sheet S1.

**Table 1:** Gene-specific primers used for tissue expression analyses and site-directed mutagenesis key amino acid sites of GmolCSP8.

Primer name	Sequence (5′→3′)	Product size (bp)
**For cloning ORF**		
Sense	CGAGGATGAAGACCATTCTG	365
Antisense	TTAGTTGCGCAGGAATTGTTC	
**For detecting the tissue expression levels**		
Sense	TGATAGCGTGTTTGTTCGCC	105
Antisense	ACCAACAACCTCTCGTTCCC	
β-actin-Sense	CTTTCACCACCACCGCTG	222
β-actin-Antisense	CGCAAGATTCCATACCCA
**For heterologous expression**		
Sense	CGGGATCCGAGACCAAATACGACT	315
	CGTC (BamHI)	
Antisense	CCCAAGCTTGGGTTAGTTGCGCAG	
	GAATTGTTC (HindIII)	
**For Site-directed mutagenesis**		
T27 → A27: ACT → GCT		
T27A-Sense	ACAGAGGGCCGTGCACACCGGAG	2998
T27A-Antisense	CGACCAAACACTTAG**C**ATAGGAC	
L30 → A30: TTG → GCG		
L30A-Sense	CTATACTAAGTGT**GC**GGTCGACAG	2998
L30A-Antisense	GACACCAACAACCTCTCGTTCCCT	
V40 → A40: GTT → GCT		
V40A-Sense	CACACCGGAGG**C**TAAACAGCTGAAG	2998
V40A-Antisense	CACGGCCCTCTGTCGACCAAACAC	
L80 → A80: TTG → GCG		
L80A-Sense	GAAACATCCAGAC**GC**GTGGAAGCA	2998
L80A-Antisense	TCCTTCAGCTCTTTGACTAGCTGC	

*The restriction endonucleases are in parentheses after each primer, and the restriction sites are underlined. Mutated nucleotide sites are highlighted in bold fonts.*

### Expression Analysis Using qRT-PCR

The relative transcriptional levels of *GmolCSP*8 in different tissues were assessed by using qRT-PCR on a CFX96 Real-Time PCR Detection System (Bio-Rad, United States). All qRT-PCR assays were conducted according to the Minimum Information for Publication of Quantitative Real-Time PCR Experiments (MIQE) Guidelines (Bustin et al., 2009). The reference gene β-actin (GenBank No: KF022227.1) of *G. molesta* was used as the endogenous control to normalize target gene expression and correct for samples. The stability of β-actin gene expression had been verified in different temperatures and tissues (Chen et al., 2014, 2018). Specific-primers for target and reference genes were designed using the online program Primer3-blast^3^ (Ye et al., 2012) and are listed in Table 1. The amplification efficiencies of target and reference genes were validated by analyzing standard curves with a 5-fold cDNA serial dilution of a female antennae template (three replicates), with resultant efficiency of 90–105%. The specificity of each primer was assessed by melting-curve analysis, which confirmed that no non-specific product was produced. To check reproducibility, each sample was analyzed using three technical and three biological replicates. Each qPCR was performed in a 20 μL reaction volume contained 10 μL of 2 × SYBR^®^ Premix Ex Taq^TM^ II mixture (TaKaRa, Dalian, China), 0.8 μL of each primer (10 μM), 2.0 μL of sample cDNA, and 6.4 μL of sterilized ddH_2_O. Samples without template cDNA served as negative controls. The qPCR cycling conditions consisted of an initial denaturing cycle of 95°C for 30 s, followed by 40 cycles of 95°C for 10 s, 60°C for 30 s and 72°C for 30 s. The relative expression of *GmolCSP*8 was quantified using the comparative 2^-ΔΔCt^ method (Livak and Schmittgen, 2001). To estimate the relative change in expression between different tissues, the abdomen sample was used as a calibrator for comparison between tissues. Significant differences in expression between tissues were assessed through one-way analysis of variance (ANOVA) (Tukey’s HSD tests) with a critical level of α = 0.05. Differences between male and female moths were measured by a paired *t*-test. All data were analyzed by using the SPSS 18.0 software (SPSS Inc., Chicago, IL, United States).

### Production and Purification of rGmolCSP8

Specific primers with a Bam HI restriction enzyme site in the sense primer and a Hind III site in the antisense primer (Table 1) were used to amplify cDNA encoding the mature GmolCSP8 (without signal peptide). The PCR product was first ligated into the pMD^®^19-T vector (TaKaRa Co., Dalian, China) by T/A Pairing, and the resulting construct was transformed into DH5α competent cells. Positive clones were grown in LB liquid medium with ampicillin (10 mg/mL) and confirmed by sequencing. pMD^®^19-T plasmids containing the target sequences were digested with Bam HI and Hind III restriction enzymes for 4 h at 37°C. The digestion products were gel-purified with a DNA Purification Kit (TianGen, Beijing, China) on agarose gels, and then ligated into the pET28a (+) (Novagen, Madison, WI, United States) expression vector with T4 DNA Ligase (Thermo Fisher Scientific, Waltham, MA, United States), previously linearized with the same restriction endonucleases. The correct plasmids containing *GmolCSP*8 genes were transferred to BL21 (DE3) competent cells for overexpression. Single colonies were grown in LB liquid medium containing 50 μg/mL of kanamycin with shaking at 220 rpm for 14 h at 37°C. The culture was diluted 1:100 with fresh LB medium and incubated under the same conditions until the OD_600_ reached approximately 0.6–0.8. Expression was induced by adding 100 mM stock solution of isopropyl-β-D-thiogalactoside (IPTG) to a final concentration of 0.5 mM for an additional 10 h at 28°C and 160 rpm. The bacterial cells were harvested by centrifugation at 8,000 g for 10 min and resuspended in lysis buffer (20 mM Tris–HCl pH 7.4, 1 mM phenylmethanesulfonyl fluoride, and 250 mM NaCl). Bacterial cells were lysed with lysozyme (10 mg/mL) for 30 min at room temperature. The cultures was sonicated and centrifuged again at 13000 g for 30 min. SDS-PAGE analysis revealed the presence of soluble rGmolCSP8, and the supernatant was enriched by complete His-Tag Purification Resin (Roche, Mannheim, Germany). Purified rGmolCSP8 was desalted through extensive dialysis with 20 mM Tris–HCl buffer (pH 7.4) until the imidazole was eliminated. The fusion tag of the pET28a (+) expression vector, which is located on the N-terminus of GmolCSP8, was not removed owing to its small size and its lack of influence on the binding-ligand ability (Chen et al., 2018; Cui et al., 2018). Concentration of soluble protein was quantified as recommended in the BCA Protein Assay Kit (Beyotime, Shanghai, China). Purified rGmolCSP8 was identified an anti-His tag monoclonal antibody (Catalog # CW0286) (Cwbio, Beijing, China) via western blot analysis, as previously described (Chen et al., 2018; Li et al., 2018).

### Fluorescence Competitive Binding Assays

To determine whether GmolCSP8 is involved in odorant binding, fluorescence competitive binding assays were performed (Zhong et al., 2012). The fluorescence spectra were recorded using a fluorescence spectrophotometer (F-4500, Hitachi, Tokyo, Japan) in a 1 cm light path fluorimeter quartz cuvette with 10 nm slits for excitation and 20 nm slits for emission. The selected chemicals are listed in Table 2, and included four sex pheromone components and 34 volatiles from different host plants (peach and pear trees) of *G. molesta* based on previous investigations (Natale et al., 2004; Lu et al., 2012; Najar-Rodriguez et al., 2013b). The fluorescent probe N-phenyl-1-naphthylamine (1-NPN) and all putative ligands used in binding assays were dissolved in methanol (HPLC purity grade) to obtain a 1 mM stock solution. The rGmolCSP8 protein was diluted to in 20 mM Tris–HCl to obtain a 2 μM solution, pH 7.4. To measure the binding affinity of 1-NPN for GmolCSP8, a 2 μM solution of protein was titrated with aliquots of 1 mM 1-NPN prepared to final concentrations ranging from 1 to 22 μM. The fluorescent probe was excited at 337 nm and emission spectra were recorded from 370 to 550 nm. The affinities of selected ligands were measured with the protein and 1-NPN at 2 μM, and the sex pheromone components were added to final concentrations of 0, 1, 2, 4, 6, 8, 10, and 12 μM, and of synthetic plant volatiles to final concentrations of 0, 2, 4, 6, 8, 12, 16, and 24 μM. Binding data were obtained from three replicates assays for each concentration of each tested ligand. Dissociation constants (*K*_d_) of protein to 1-NPN were calculated by non-linear regression, using GraphPad Prism 4.00 software (GraphPad Software Inc.). The inhibition constants (*K*_i_) of the competitors were determined using the corresponding IC50 values (concentration of competitor that reduced the initial fluorescence intensity by half) based on the following equation: *K_i_* = [IC_50_]/(1+[1-NPN]/K*_1-NPN_*), where [1-NPN] is the free concentration of 1-NPN and K_1-NPN_ is the dissociation constant of the complex GmolCSP8/1-NPN. In this study, if the IC_50_ value was less than 10 μM, the tested ligands were considered to exhibit strong binding affinities to rGmolCSP8; an IC_50_ value between 10 and 20 μM indicated that ligands demonstrated medium binding ability to protein, an IC_50_ exceeding 20 μM indicated that ligands have a weak binding capacity to protein. If the IC_50_ value exceeded 24 μM, no further calculation of the binding affinity was considered.

**Table 2:** Binding affinities of rGmolCSP8 to sex pheromone and host-plant volatile compounds were detected via fluorescence binding assays.

Chemical compounds	Nature source of ligand	Source/Purity (Synthetics)	IC_50_	K*_i_*
(*Z*)-8-dodecenyl acetate	Sex pheromone component	Bedoukian Research, > 95.0%(AR),	>24	>24
(*E*)-8-dodecenyl acetate	Sex pheromone component	Bedoukian Research, > 95.0%(AR)	>24	>24
(Z)-8-dodecenyl alcohol	Sex pheromone component	Bedoukian Research, > 98.0%(AR)	>24	>24
1-dodecanol	Sex pheromone component	aladdin, > 99.0%(GC)	13.54 ± 0.83	12.90 ± 0.56
1-butanol	Pear fruit volatile	aladdin, ≥ 99.9%(GC)	7.32 ± 0.56	6.65 ± 0.52
1-pentanol	Peach and pear fruit volatile	aladdin, ≥ 99.9%(GC)	8.79 ± 0.99	8.18 ± 0.93
3-methyl-1-butanol	Pear fruit volatile	TCI, > 98.0%(AR)	6.60 ± 0.45	6.32 ± 0.52
1-hexanol	Green volatile	TCI, > 98.0%(AR)	2.26 ± 0.34	2.11+0.32
*Cis*-3-hexen-1-ol	Green volatile	Sigma, 98.0%(AR)	9.14 ± 0.73	8.51 ± 0.68
1-heptanol	Peach shoot volatile	aladdin, ≥ 99.5%(GC)	6.79 ± 0.56	6.20 ± 0.42
1-decanol	Peach shoot volatile	aladdin, ≥ 99.5%(GC)	7.16 ± 0.58	6.60 ± 0.54
1-tetradecanol	Pear flower volatile	aladdin, 97.0%(AR)	>24	>24
(*E*)-2-hexenal	Green volatile	Alfa, 98.0%(AR)	>24	>24
Hexanal	Green volatile	TCI, > 95.0%(AR)	>24	>24
Benzaldehyde	Peach flower and pear fruit volatile	aladdin, ≥ 99.5%(GC)	>24	>24
Heptanal	Peach flower and pear fruit volatile	aladdin, 97.0%(AR)	>24	>24
Octanal	Peach flower and pear fruit volatile	aladdin, 99.0%(AR)	>24	>24
Nonanal	Peach flower and pear fruit volatile	Sigma, 95.0%(AR)	>24	>24
Decanal	Pear fruit volatile	aladdin, 97.0%(AR)	>24	>24
Ethyl butyrate	Pear fruit volatile	aladdin, 99.0%(AR)	>24	>24
Butyl acetate	Pear fruit volatile	aladdin, 99.0%(AR)	>24	>24
Isoamyl acetate	Peach and pear fruit volatile	aladdin, ≥ 99.5%(GC)	>24	>24
*Cis*-3-hexenyl acetate	Green volatile	TCI, > 97.0%(GC)	>24	>24
Butyl butyrate	Pear fruit volatile	aladdin, > 99.0%(GC)	>24	>24
Ethyl hexanoate	Pear fruit volatile	aladdin, 99.0%(AR)	>24	>24
Hexyl acetate	Pear fruit volatile	aladdin, 99.0%(AR)	>24	>24
Methyl salicylate	Peach shoot and pear fruit volatile	Sigma, ≥ 99.0%(GC)	>24	>24
Ethyl heptanoate	Peach fruit volatile	aladdin, ≥ 99.5%(GC)	>24	>24
Butyl hexanoate	Pear fruit volatile	aladdin, ≥ 99.5%(GC)	>24	>24
Methyl jasmonate	Peach fruit volatile	aladdin, 98.0%(AR)	>24	>24
α-Pinene	Peach and pear tree volatile	Sigma, 98.0%(AR)	>24	>24
α-Ocimene	Peach and pear tree volatile	aladdin, ≥ 90.0%(AR)	18.85 ± 1.29	17.58 ± 0.81
Benzonitrile	Peach shoot volatile	TCI, > 99.0%(AR)	>24	>24
Decane	Pear fruit volatile	aladdin, 98.0%(AR)	>24	>24
Tetradecane	Peach and pear fruit volatile	aladdin, 98.0%(AR)	>24	>24
Pentadecane	Peach and pear fruit volatile	aladdin, 98.0%(AR)	>24	>24
Hexadecane	Peach and pear fruit volatile	aladdin, 98.0%(AR)	>24	>24
Octadecane	Peach and pear fruit volatile	aladdin, 98.0%(AR)	>24	>24

*More than > 24 μM indicate that the IC_50_ and K_i_ values are out of concentration ranges tested.*

### Effect of pH on the Binding Affinity of rGmolCSP8

Based on the fluorescent binding assays described above, we further assessed the effects of pH on the binding abilities of GmolCSP8 with strongly bound ligand. The binding affinities of rGmolCSP8 to the representative ligand 1- hexanol were measured at six different pH values (4.0, 5.0, 6.8, 7.4, 8.0, and 8.8) in 20 mM Tris–HCl buffer. pH was adjusted by titrating concentrated hydrochloric acid using a PHS-3E pH meter (Shanghai Precision Scientific Instrument Co., Ltd., Shanghai, China). The binding assays and data processing were performed as described previously.

### Three-Dimensional (3D) Structure Modeling and Molecular Docking of Ligand

A protein blast search was performed for the GmolCSP8 amino acid sequence against the Protein Data Bank (PDB) to identify structural templates for homology modeling. Based on sequence similarity and coverage of the target and template proteins (Supplementary Table S1), we selected the crystal structure of CSPsg4 from the desert locust *Schistocerca gregaria* (PDB ID: 2gvs.1) as a template to generate the 3D structure of GmolCSP8 using Modeller 9.10 software (Gunda et al., 2016) with an automated approach. A 1-hexanol model was generated using ChemBioDraw12.0 (Cousins, 2011), and the three-dimensional structure was preserved after optimization via MM2 energy minimization. The molecular docking of GmolCSP8 binding 1-hexanol was performed using Autodock 4.0 software tool as the following methods: first, the structures of 1-hexanol and 3D models of GmolCSP8 were optimized by adding hydrogens using Kollman charges. Then, the model was prepared by optimizing torsion angles and saved in PDBQT format. Finally, flexible docking of GmolCSP8 with 1-hexanol was performed by Autodock software, without considering the effect of water and other solvents. Amino acids of 3Å around 1-hexanol were considered to be important residues of the binding ligand.

### Site-Directed Mutagenesis and Expression of Mutants

To verify the four predicted key amino acid sites of GmolCSP8 binding for 1-hexanol, recombinant plasmids for four CSP8 mutants were obtained via site-directed mutagenesis experiments. Specific primers introducing mutation sites of the four key amino acid CSP8 plasmids (GmolCSP8-T27A, GmolCSP8-L30A, GmolCSP8-V40A, and GmolCSP8-L80A) were designed and are listed in Table 1. The four mutants were mutated by using the TaKaRa MutanBEST Kit (TaKaRa, Shiga, Japan) with the pMD^®^19-T/GmolCSP8 plasmid DNA as template. All plasmids containing mutations were validated by sequencing with five randomly selected single colonies. pMD^®^19-T plasmids including four CSP8 mutants were digested with Bam HI and Hind III restriction enzymes. Then, the target sequences were ligated into the expression vector pET28a (+) and transformed into BL21 (DE3) competent cells for expression. After sequencing verification, the corresponding rGmolCSP8 mutant proteins were induced and purified, and the binding affinities to 1-hexanol and its analogs were verified via ligand-binding assays according to the method described above.

## Results

### Cloning and Phylogenetic Analysis of GmolCSP8

**FIGURE 1:**
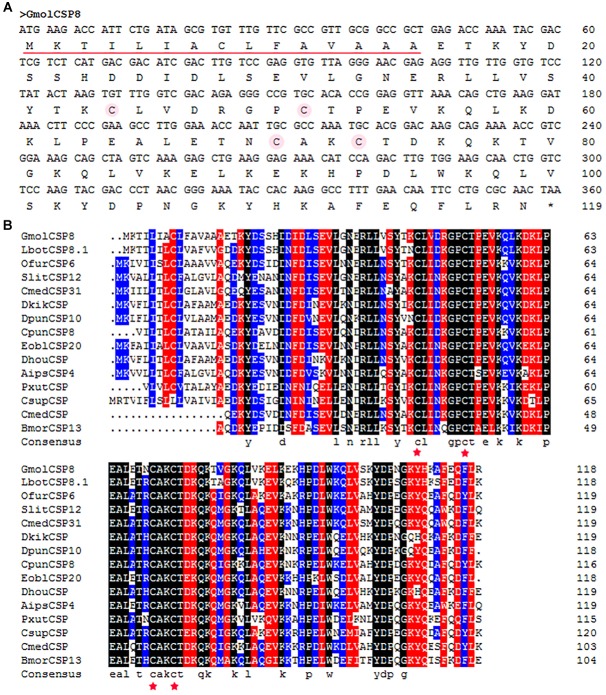
Cloning and phylogenetic analysis of GmolCSP8 from *Grapholita molesta*. **(A)** Nucleotide and deduced amino acid sequence of GmolCSP8 from *G. molesta*. The four conserved cysteines are marked by an amaranthine circle. The predicted signal peptide sequence is underlined. **(B)** Sequence alignment of GmolCSP8 with other insect species. The four conserved cysteines are marked with five-pointed red star. The names and the GenBank accession number of the 15 CSPs are as follows: GmolCSP8, *Grapholita molesta*, ALC79595.1; LbotCSP8.1, *Lobesia botrana*, AXF48705.1; OfurCSP6, *Ostrinia furnacalis*, BAV56810.1; SlitCSP12, *Spodoptera litura*, ALJ30223.1; CmedCSP31, *Cnaphalocrocis medinalis*, ALT31613.1; DkikCSP, *Dendrolimus kikuchii*, AII01029.1; DpunCSP10, *Dendrolimus punctatus*, ARO70314.1; CpunCSP8, *Conogethes punctiferalis*, AHX37222.1; EoblCSP20, *Ectropis obliqua*, ALS03845.1; DhouCSP, *Dendrolimus houi*, AII01021.1; AipsCSP4, *Agrotis ipsilon*, AGR39574.1; PxutCSP, *Papilio xuthus*, BAF91717.1; CsupCSP, *Chilo suppressalis*, AHC05674.1; CmedCSP, *Cnaphalocrocis medinalis*, AIX97832.1; BmorCSP13, *Bombyx mori*, NP_001037180.1.

The full-length cDNA of GmolCSP8 was cloned and then confirmed by sequencing. Three incorrect basic groups were identified in the Unigene of GmolCSP8, obtained by antennal transcriptome sequencing, and the verified sequence was deposited in GenBank under accession number KR003781. GmolCSP8, encoded by 119 amino acids with an open reading frame (ORF) of 360 bp, was found to contain a predicted signal peptide of 15 amino acid residues at the N-terminus (Figure 1A). The calculated molecular weight (MW) and isoelectric point (pI) of mature GmolCSP8 is 13.53 kDa and 8.22, respectively.

**FIGURE 2:**
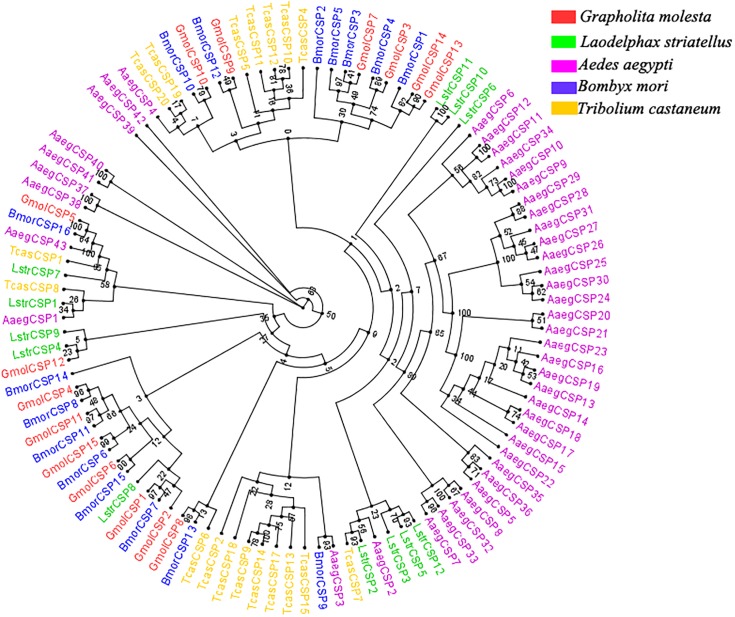
Phylogenetic tree of CSPs from *Grapholita molesta* and other insects through the Neighbor-joining method with a bootstrap replication of 1,000. The GenBank accession number and the corresponding amino acid sequences used in the phylogenetic analyses were listed in Supplementary Data Sheet S1. The unrooted tree was constructed using the BioNJ algorithm in Seaview v.4.0 based on the sequence alignment produced via MAFFT version 6. Gmol, *Graphilita molesta*; Lstr, *Laodelphax striatella*; Cqui, *Culex quinquefasciatus*; Bmor, *Bombyx mori*; Tcas, *Tribolium castaneum*.

Moreover, multiple sequence alignment of GmolCSP8 with homologous CSPs from other Lepidopteran species revealed the presence of a typical conserved motif containing four cysteines (C_1_-X_6-8_-C_2_-X_16-21_-C_3_-X_2_-C_4_, X represent any amino acids except cysteine) (Figure 1B). Furthermore, using the NCBI BLASTx program, we found that GmolCSP8 shared relatively high sequence similarity with other insect CSPs, with 82% identity with *Lobesia botrana* LbotCSP8 (GenBank No. AXF48705.1), and almost 60% identities with *Ostrinia furnacalis* OfurCSP6 (GenBank No. BAV56810.1), *Spodoptera litura* SlitCSP12 (GenBank No. ALJ30223.1), *Cnaphalocrocis medinalis* CmedCSP31, *Dendrolimus punctatus* DpunCSP10 (GenBank No. ARO70314.1), *Conogethes punctiferalis* CpunCSP8 (GenBank No. AHX37222.1), and *Ectropis obliqua* EoblCSP20 (GenBank No. ALS03845.1). GmolCSP8 shared relatively low sequence identity (less than 50%) with the other 14 CSPs from intraspecific species (Supplementary Table S2). Subsequently, 104 CSPs obtained from different order insects comprised of *Graphilita molesta* (15 sequences), *Laodelphax striatella* (12 sequences), *Aedes aegypti* (43 sequences), *Bombyx mori* (16 sequences) and *Tribolium castaneum* (18 sequences) were selected and used to construct phylogenetic tree. Phylogenetic analysis showed that GmolCSP8 was clustered into a small branch close to BmorCSP13 from *B. mori* and TcasCSP6 from *T*. *castaneum* (Figure 2). The majority of CSPs of *A. aegypti* and *T*. *castaneum* clustered into the same branch with intraspecific CSPs, while the CSPs of *G*. *molesta*, *B*. *mori*, and *L*. *striatella* were highly divergent and clustered into dozens of branches of the in Neighbor-joining tree (Figure 2).

### Tissue Expression of *GmolCSP*8

**FIGURE 3:**
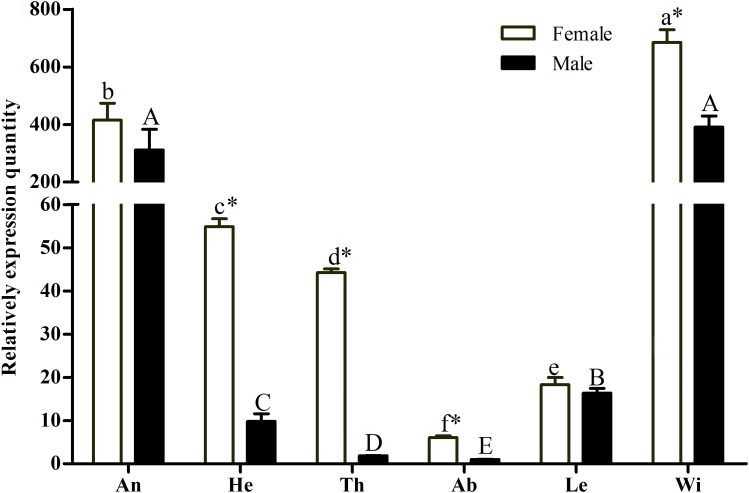
Relative expression level of *GmolCSP*8 in different tissues of *Grapholita molesta*. An: antennae, He: heads, Th: thoraxes, Ab: abdomen, Le: legs, Wi: wings. The fold changes are relative to the expression levels in the abdomen. Different lowercase and capital letters above each bar indicate significantly differences (*P < 0.05*, ANOVA, Tukey) among different tissues in female and male, respectively. ^∗^Indicate significant differences in expression levels between two sexes in the same tissues (Independent *t*-test, *α* = 0.05).

A pilot experiment showed that the amplification efficiencies of target and reference genes were between 90 and 105%, with no non-specific amplification (Supplementary Figure S1), confirming that the two primer pairs and selected amplification conditions could be used for subsequent quantification. The qRT-PCR results showed that *GmolCSP*8 was predominantly expressed in the wings and antennae of both males and females, but lowly expressed in abdomen and legs of both males and females, as well as in heads and thoraxes of males (Figure 3). In addition, the expression of *GmolCSP*8 in the wings, heads, thoraxes and abdomen differed significantly between males and females, with 5.60- and 23.48-fold higher expression in the heads and thoraxes of female than those of male. It is speculated that *GmolCSP*8 may participate in chemoreception and other physiological functions based on its expression characteristics.

### Fluorescent Binding Assays

**FIGURE 4:**
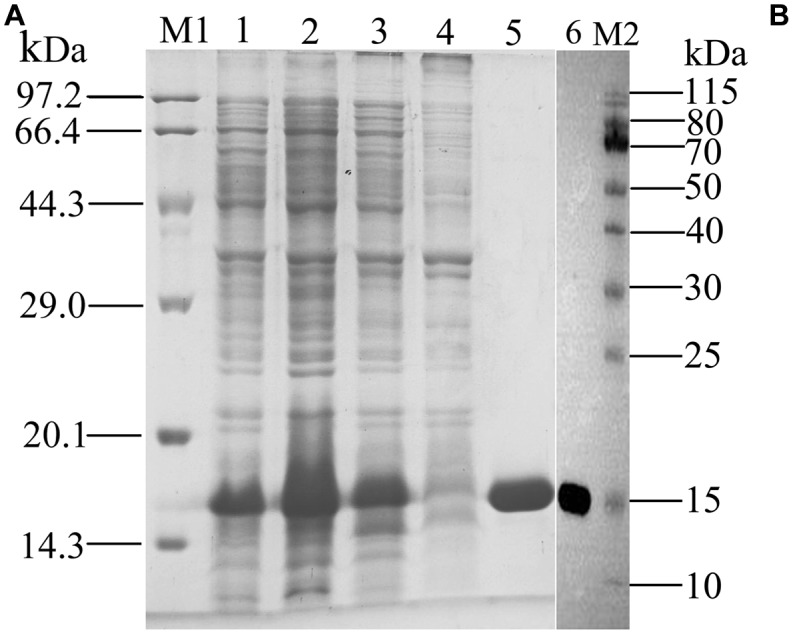
SDS-PAGE **(A)** and Western blot **(B)** analysis of the expressed products and purified of rGmolCSP8. M1, M2: Protein molecular weight marker; 1. Non-induced pET28a(+)/GmolCSP8; 2. Induced pET28a(+)/GmolCSP8; 3. pET28a(+)/GmolCSP8 supernatant; 4. pET28a(+)/GmolCSP8 pellet; 5. Purified pET28a (+)/GmolCSP8; 6. Western Blot analysis of purified rGmolCSP8.

**FIGURE 5:**
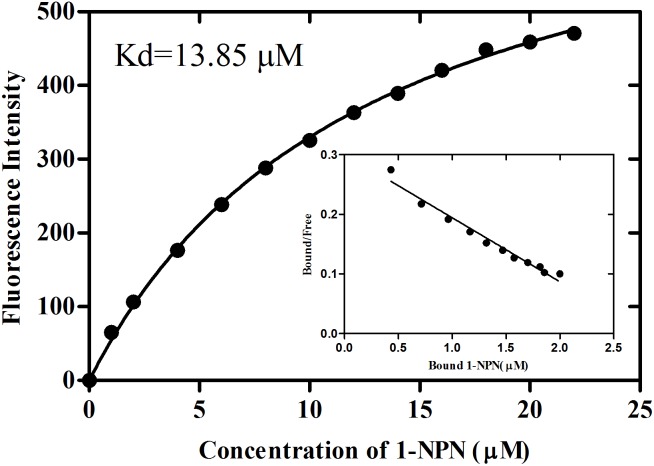
Binding curve and relative Scatchard plot of 1-NPN to rGmolCSP8. A 2 μM of rGmolCSP8 protein in 20 mM Tris–HCl buffer (pH 7.4) was titrated with aliquots of 1 mM 1-NPN stock solution to final concentrations of 0 to 22 μM, and the emission spectra were recorded between 370 and 550 nm. The calculated dissociation constant of rGmolCSP8 was 13.85 μM.

**FIGURE 6:**
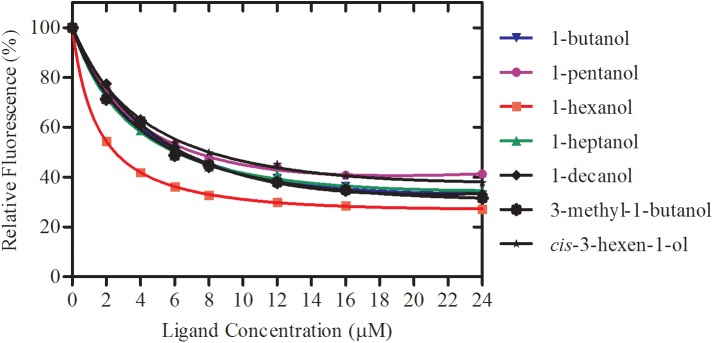
Binding curves of rGmolCSP8 with the small aliphatic alcohols. Mixtures of 2 μM rGmolCSP8 and 2 μM 1-NPN were titrated with 1 mM stock solution of each ligand to final concentrations of 0–24 μM. Relative fluorescence intensities are reported as percentages of the initial fluorescence values without competitors.

rGmolCSP8 was successfully expressed in soluble forms in a prokaryotic expression system with a high yield (about 0.8 mg/mL). Subsequently, the expression and purification of rGmolCSP8 was examined by 15% SDS-PAGE (Figure 4A) and western blot (Figure 4B), respectively. In addition, the binding affinities of GmolCSP8 to various ligands were determined by ligand-binding assays. Among those, binding of the fluorescent probe 1-NPN to GmolCSP8 was evaluated and a gradual saturation and linear Scatchard plot was obtained, yielding a *K*_d_ value of 1-NPN with rGmolCSP8 of 13.85 μM (Figure 5). In summary, 38 chemicals were selected as putative ligands to detect the binding characteristics of rGmolCSP8, which was comprised of four sex pheromones and 34 volatiles from peach and pear trees (Table 2). The results showed that rGmolCSP8 exhibited specific binding properties and could only bind to a few ligands, such as alcohols and terpenes. Of those, rGmolCSP8 showed medium binding affinity to the minor sex pheromone component 1-dodecanol (12:OH) with a *K*_i_ value of 12.90 μM. In addition, rGmolCSP8 displayed the strongest binding affinities to green leaf volatile 1-hexanol with a *K*_i_ value of 2.11 μM, as well as the other small aliphatic alcohols comprised of 1-butanol (6.65 μM), 1-pentanol (8.18 μM), 3-methyl-1-butanol (6.32 μM), *cis*-3-hexen-1-ol (8.51 μM), 1-heptanol (6.20 μM), and 1-decanol (6.60 μM), respectively (Figure 6). However, for α-ocimene, rGmolCSP8 showed a weak binding affinity with a *K*_i_ value of 17.58 μM. However, rGmolCSP8 did not bind to the tested aldehydes, esters, benzonitriles, or alkanes (Table 2).

### Effect of pH on the Binding Affinity of rGmolCSP8

**FIGURE 7:**
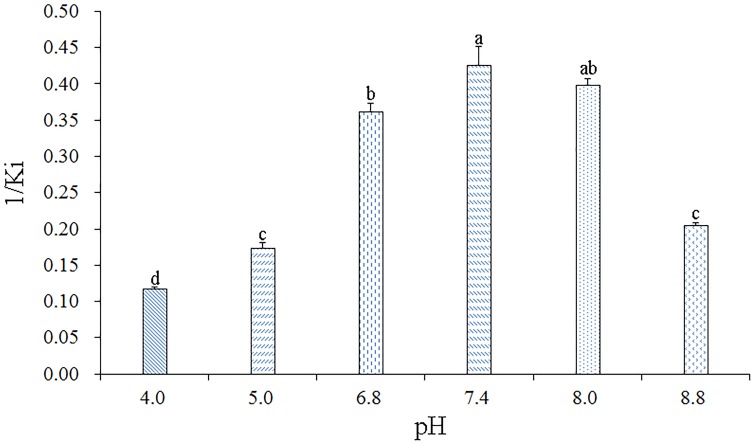
Effects of pH on the binding affinities of rGmolCSP8 with 1-hexanol. The mixture solution of 2 μM rGmolCSP8 protein and 2 μM 1-NPN was titrated with 1 mM 1-hexanol stock solution to final concentrations of 22 μM. All data represent a mean of three independent measurements, and the bars denote mean ± SEM.

To determine the effect of pH on the binding affinity of rGmolCSP8 to ligand, the binding abilities of GmolCSP8 in different pH buffered solutions (20 mM Tris–HCl) were evaluated with the representative ligand 1-hexanol *in vitro*. The resulting binding values indicated that pH significantly affected the binding affinities of rGmolCSP8 to 1-hexanol; the binding affinity was higher at pH 6.8–8.0 than at pH 4.0–5.0 and 8.8 (Figure 7), revealing that ligand binding and protein release were related to a pH-dependent conformational transition.

### Structural Modeling and Molecular Docking

**FIGURE 8:**
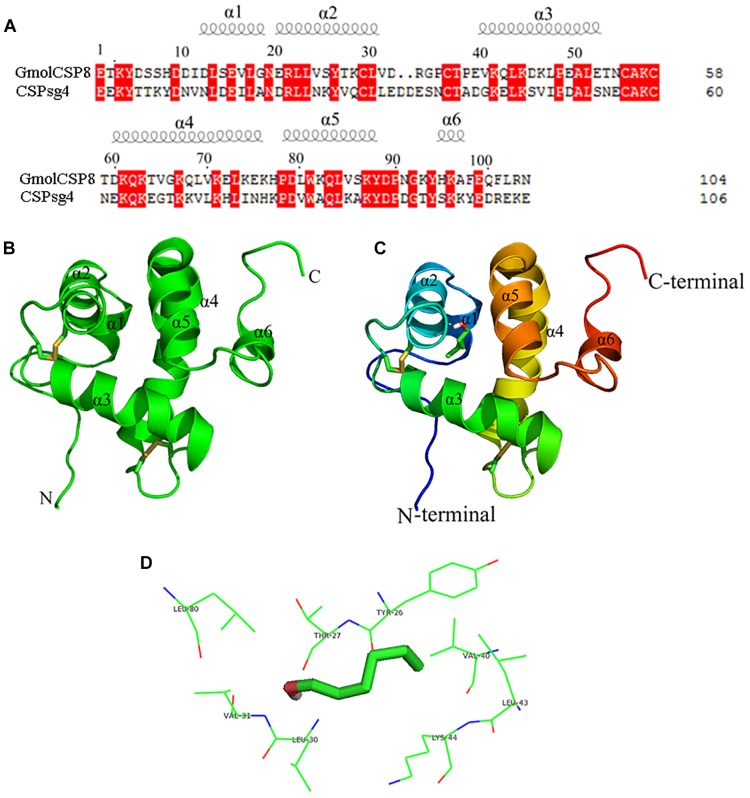
Modeled three-dimensional structure and molecular docking of GmolCSP8. **(A)** Sequence alignment of GmolCSP8 and CSPsg4 (2gvs.1). α-helices are displayed as spiral coils. The same residues are highlighted with a red background. **(B)** Three-dimensional structure of GmolCSP8. Two disulfide bridges are colored in yellow. α-helices, N-terminal, and C-terminal are labeled. **(C)** Molecular docking of GmolCSP8 with 1-hexanol. 1-hexanol is displayed as a stick model with the hydroxyl oxygen in red. **(D)** The interaction diagram of amino acid residues to 1- hexanol. The top eight potential key residues (Tyr26, Thr27, Val40, Leu43, Lys44, Leu30, Val31, and Leu80) are shown with black font.

Structural templates for GmolCSP8 from *G. molesta* were identified by sequence alignments. The results demonstrated that GmolCSP8 shared more than 30% sequence identities with four structurally determined CSPs, including *Schistocerca gregaria* CSPsg4 (2gvs.1), *Mamestra brassica*e MbraCSP2 (1k19.1.A), *M. brassicae* MbraCSPA6 (1kx9.1.A) and *Bombyx mori* BmorCSP1 (2jnt.1.A). Importantly, GmolCSP8 was found to share the highest similarity and coverage with CSPsg4, with values of 44.7 and 95.0%, respectively (Figure 8A). Finally, the 3D structure of GmolCSP8 was generated using the CSPsg4 structure as a template. The generated structure was validated by PROCHECK. The model presented 89.2% of amino acid residues in the core region, 8.6% of amino acid residues in the additionally allowed region, 1.1% of amino acid residues in the generously favored region, and 1.1% of amino acid residues in the disallowed region. A Ramachandran plot of the modeled protein is shown in Supplementary Figure S2.

The simulated 3D structure of GmolCSP8 had an overall globular shape and consisted of six α-helices, represented as α1 (Leu13-Gly18), α2 (Glu20-Val31), α3 (Val40-Glu52), α4 (Asp60-Lys76), α5 (Pro78-Tyr88), and α6 (His95-Ala97). Of those, helices α1 and α2 (residues 13–31), together with helices α4 and α5 (residues 60–88) formed two paralleled V-shaped structures; and helix α3 was perpendicular to the planes determined by two V-shaped structures and positioned between the four ends of the two V-shaped structures (Figure 8B). In addition, helix α6 was located on the outer surface of the α4–α5 helices and did not participate in the formation of the binding pocket. Four conserved cysteines are linked, in turn, to form two pairs of disulfide bridges (connected Cys29 and Cys36, Cys55, and Cys58) to maintain the spatial structure of protein. The residue Cys29 is located on the helices, and the residues Cys36, Cys55, and Cys58 are located on loops.

Molecular flexible docking was conducted to predict the key binding sites of GmolCSP8 to 1-hexanol (Figure 8C). Interactions between functional groups of ligand and binding sites of GmolCSP8 are shown in Figure 8D. The docking simulation showed that Tyr26, Thr27, Val40, Leu43, Lys44, Leu30, Val31, and Leu80 may be responsible for the binding to 1-hexanol and Thr27 might be involved in specific recognition via H-bonding with hydroxyl oxygen of 1-hexanol. To determine whether each predicted residue had an important role in 1-hexanol binding, four residues (Thr27, Leu30, Val40, and Leu80) with lower interaction energy to 1-hexanol were mutated to alanine using site-directed mutagenesis. Then, the functions of putative key residues were validated by measuring the binding affinity of each mutant protein to 1-hexanol.

### Site-Directed Mutagenesis of GmolCSP8 and Binding Affinities of Mutants

**FIGURE 9:**
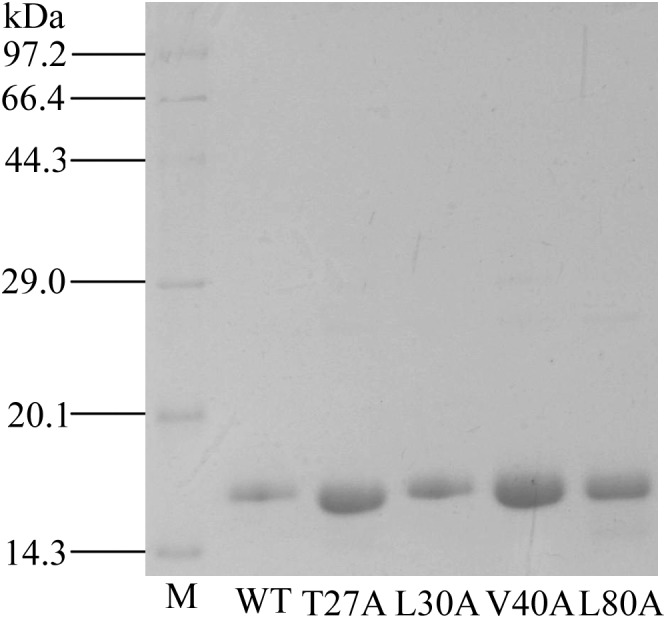
SDS-PAGE analysis of rGmolCSP8 wild-type (WT) and mutants T27A, L30A, V40A, and L80A. M represents protein molecular weight marker.

**FIGURE 10:**
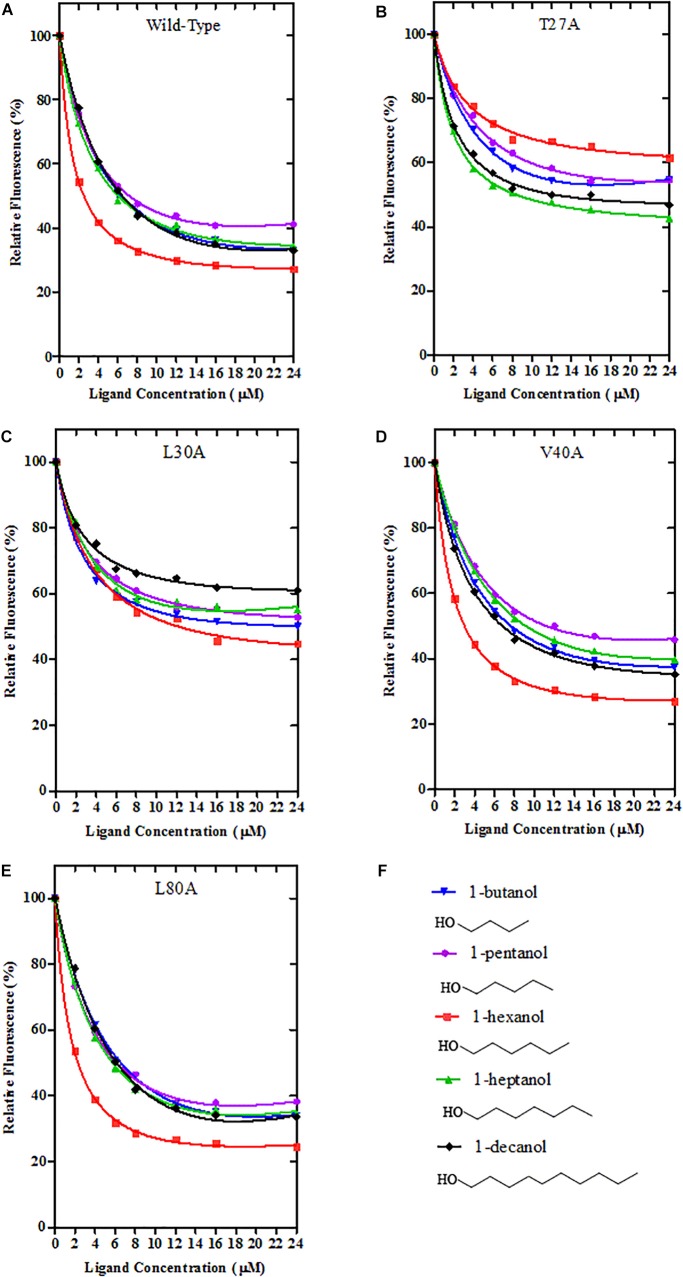
Binding curves of Wild-Type GmolCSP8 and select mutants. **(A)** Wild-Type GmolCSP8, **(B)** mutant T27A, **(C)** mutant L30A, **(D)** mutant V40A, **(E)** mutant L80A, and **(F)** ligand compounds and their structural formulas. The mixture solution of 2 μM each recombinant protein and 2 μM 1-NPN was titrated with 1 mM ligand stock solution to final concentrations of 2 μM. Relative fluorescence intensities are reported as percentages of the initial fluorescence values without competitors.

Using site-directed mutagenesis, four predicted key amino acid residues (Thr27, Leu30, Val40, and Leu80) of GmolCSP8 were substituted with alanine (Ala), yielding the four mutants T27A, L30A, V40A, and L80A (Figure 9). There was no significant difference in the calculated dissociation constant (*K*_d_) of the four mutants to the fluorescence probe 1-NPN (Supplementary Figure S3). To evaluate the functions of these residues in the binding process, the binding affinities of mutant CSP8-T27A, CSP8-L30A, CSP8-V40A, and CSP8-L80A proteins to 1-hexanol and its analogs (Figure 10F) were measured using binding assays (Table 3). Compared with wild-type rGmolCSP8, the ability of CSP8-T27A to bind to 1-hexanol, 1-butanol, and 1-pentanol were significantly reduced and did not displace 50% 1-NPN from the CSP8-T27A/1-NPN complex solution at concentrations of ligands up to 24 μM. It also demonstrated a 1.5- to 2.0-fold decrease toward 1-heptanol and 1-decanol, indicating the Thr27 was one of the key amino acid residues of GmolCSP8 involved in the binding to 1-hexanol, Thr27 may engage in H-bond with this ligand (Figure 10A,B). Mutant CSP8-L30A lost its ability to bind to 1-pentanol, 1-heptanol, and 1-decanol and demonstrated a 6-fold decrease in its ability to bind 1-hexanol (Figure 10C), revealing that Leu30 is an important residue in the binding site of GmolCSP8 for 1-hexanol and its homologs. The mutants CSP8-V40A and CSP8-L80A showed only a slightly change in binding to 1-hexanol and its analogs (Figure 10D,E); a possible explanation is that Val40 and Leu80 do not participate in recognition of the small aliphatic alcohols.

**Table 3:** Binding affinities of 1-hexanol and its analogs to rGmolCSP8 Wild-Type and mutants.

Ligands	*K*_i_ (μM)				
	Wild-Type	T27A	L30A	V40A	L80A
1-butanol	6.65 ± 0.52	–	17.09 ± 1.05	8.07 ± 0.61	6.75 ± 0.57
1-pentanol	8.18 ± 0.93	–	–	12.36 ± 1.41	6.66 ± 0.64
1-hexanol	2.11+0.32	–	12.51 ± 1.12	2.67 ± 0.34	1.80 ± 0.15
1-heptanol	6.32 ± 0.52	9.01 ± 0.90	–	9.68 ± 0.75	6.02 ± 0.66
1-decanol	6.67 ± 0.54	12.67 ± 1.79	–	7.02 ± 0.47	6.32 ± 0.65

*K_i_, dissociation constant. The values are the means of three independent experiments. “–” means the IC_50_ and K_i_ values were not calculated in the range of concentration ranges of tested ligands (up to 24 μM).*

## Discussion

Analysis of the tissue expression profiles of CSP genes can help to understand their function in insects. *GmolCSP*8 was highly expressed in antennae, suggesting potential roles in olfactory chemoreception. Antennae are the most important olfactory organs in insects; they function as a switch that regulates the entry of volatile semiochemicals from the outside environment into the olfactory system (Gao and Chess, 1999; Leal, 2013). Several studies have shown that antenna-specific or -highly expressed CSPs can selectively perceive and recognize sex pheromones and general odorants (Calvello et al., 2005; Peng et al., 2017). In insects, antennae, wings, and legs belong to contact organs while the head, thorax, and abdomen are classified as non-contact organs (Gong et al., 2007;He et al., 2011; Hua et al., 2013). *GmolCSP*8 was also highly expressed in wings; the relative expression in female wings was significantly higher than that in male wings, indicating that it may be involved in contact chemoreception, especially for females. The biased or high expression of CSPs in female wings has also been found in other insects; for example, *Apolygus lucorum* *AlucCSP*2 and *AlucCSP*3 were specifically expressed in female wings (Hua et al., 2013), *Nilaparvata lugens* *NlugCSP*5 was biased and highly expressed in female wings (Yang et al., 2014). The transcript level of *GmolCSP*8 in the abdomen of female and male adults was negligible; combined with the recombinant protein not binding the major sex pheromones; we speculated that this gene may not participate in the transport of sex pheromones in the gonads (Li Z.Q. et al., 2015). The ubiquitously expression of GmolCSP8 in *G. molesta* indicates that this gene may be involved in functions other than chemosensing (Guo et al., 2011; Ali et al., 2019).

To elucidate the olfactory function of GmolCSP8, 38 compounds, including four sex pheromones and 34 plant volatiles emitted from peach and pear trees, were selected for ligand-binding assays using 20 mM Tris–HCl (at pH 7.4) as a buffer solution (Cardé et al., 1975, 1979; Natale et al., 2003; Najar-Rodriguez et al., 2013b; Lu et al., 2015). GmolCSP8 exhibited specific-binding affinities to green leaf volatile 1-hexanol, as well as its analogs 1-butanol, 3-methyl-1-butanol, 1-heptanol, 1-decanol, and 1-dodecanol. Conversely, others behaviorally important ligands such as (*Z*)-3-hexenyl acetate, (*E*)-2-hexenal, hexanal, heptanal, benzaldehyde, butyl hexanoate, α-pinene and benzonitrile, cannot bind to GmolCSP8. For example, a three-compound mixture of (Z)-3-hexenyl acetate, (Z)-3-hexen-1-ol and benzaldehyde in a 4:1:1 proportion elicited a similar attractant effect on female *G*. *molesta* as the natural blend from peach shoot volatiles (Natale et al., 2003); butyl hexanoate was clearly attractive for virgin and mated female *G*. *molesta* at doses of 0.32–1.35 mg (Natale et al., 2004). Five aldehydes, including hexanal, heptanal, octanal, and nonanal, and benzaldehyde, play essential roles in female attraction to host plants (Najar-Rodriguez et al., 2013b). The special binding property with small linear aliphatic alcohols suggested that GmolCSP8 may possess a suitable binding site for ligands with a hydroxyl functional group. The length of the carbon chain significantly affects the affinity of protein with ligands; GmolCSP8 exhibited the strongest affinity to 1-hexanol (C6) with a *K*_i_ value of 2.11 μM, and also displayed relative higher binding abilities to other aliphatic alcohols with 4–12 carbon atoms with a *K*_i_ value of 6.60–12.90 μM. It is unable to bind to 1-tetradecanol when the number of carbon atoms in the ligand increases to 14. 1-dodecanol is a minor sex pheromone component of *G. molesta*, and functions as a synergist of sex attractant, which increased the frequency of male landing and as a stimulant that induces the occurrence of mating behavior when adult moths of both sexes are in close proximity (Cardé et al., 1975, 1979). The results of ligand-binding assays showed that GmolCSP8 has medium binding abilities to 1-dodecanol with a *K*_i_ value of 12.90 μM *in vitro*. The binding affinities of this protein to 1-dodecanol were significantly lower than those of GmolOBPs to 1-dodecanol (e.g., the *K*_i_ values of GmolGOBP2, GmolOBP8, and GmolOBP15 to 1-dodecanol were 1.22, 4.39, and 3.24 μM, respectively) (Li et al., 2016a,b). Further validation combining gene knockout experiments and behavioral assays is required to confirm whether GmolCSP8 is involved in the perception and recognition of 1-dodecanol.

The binding values of GmolCSP8 to its preferred ligand 1-hexanol under different pH environments revealed that a pH-dependent conformational transition is involved in binding and releasing protein to ligands. GmolCSP8 displayed a strong binding affinity to 1-hexanol at pH 6.8–8.0, while it showed a very weak affinity at pH 4.0. The binding and release mechanism of GmolCSP8 was similar to that of BmorPBP1 and BmorGOBP2 of *Bombyx mori*, ApolPBP of *Antheraea polyphemus*, and AtraPBP1 of *Amyelois transitella* (Mohanty et al., 2004; Zhou et al., 2009; Xu et al., 2010). The longer C-terminal tail of BmorPBP1 can form an additional helix, α7, which is inserted between the first and second α-helix and occupies a bombykol-binding site at low pH resulting in ligand release, The helix α7 converted to an unfolded conformation under a neutral pH value and exhibited ligand-binding activity (Sandler et al., 2000). Another explanation is that the protonation of sidechains causes a general unraveling of the BmorPBP1 conformation as the pH is reduced, and the loss of secondary structure resulted in the opening of the binding pocket to release sex pheromones (Lartigue et al., 2002). Several CSPs have demonstrated that a pH-induced conformational change triggers ligand-binding and -release, including MaltCSP5 from *Monochamus alternatus* and MsepCSP8 from *Mythimna separate* (Younas et al., 2018; Ali et al., 2019). The α-helical content of CmedCSP33 was decreased at low pH values, suggesting the protein conformation loosened, which may facilitate odorant binding (Duan et al., 2019). However, the pH-dependent mechanism of CSPs in the binding and release of odorants remains unclear. Compared with the three-dimensional structure of OBPs, five helices (α1, α2, α4, α5, and α3) in CSPs form narrower and elongated channels, which conveniently bind aliphatic ligands. In contrast to the longer C-terminal of OBPs, CSPs possess a disordered and long N-terminal; whether the N-terminal peptide segment can form a regular helix at some pH values and trigger binding and release in a comparable way to the C-terminal helix of OBP remains to be explored.

Chemosensory proteins are important odorant recognition proteins during the initial process of olfactory perception, and the detailed interactions of these proteins with specific odor molecules have been elucidated via site-directed mutagenesis (Tan et al., 2018). Several predicted key binding site residues of CSPs form a hydrogen bond, including Tyr26 of CSPMbraA6 with 12-bromo-dodecanol (Lartigue et al., 2002), Trp83 of CSPsg4 with oleamide (Tomaselli et al., 2006), and Cys 60 of Ac-ASP3 with isoamyl acetate (Liu et al., 2013). Some insect CSPs use van der Waals forces to bind odor molecules, such as Phe29 of CmedCSP33 with nerolidol (Duan et al., 2019), and F44 of AmelCSP1 to ionone (Tan et al., 2018). Compared with the wild type rGmolCSP8 protein, the T27A mutation abolished the ability to bind 1-hexanol. The most credible interpretation is that the oxygen atom of the hydroxyl group in 1-hexanol forms a hydrogen bond with Thr27; this ligand cannot be recognized by the mutant protein due to the loss of hydrogen bonding. The L30A mutant showed an approximate 6-fold decreased in binding to 1-hexanol; we speculated that the Van der Waals force formed by Leu30 of GmolCSP8 and 1-hexanol, and possibly by Thr27 and Leu30, combined to recognize 1-hexanol. V40A and L80A mutants revealed no significant change in the binding affinities to 1-hexanol and its analogs, suggesting that Val40 and L80A are not involved in the recognition of these ligands. Overall, Thr27 is the key binding site of GmolCSP8, involved in the initial recognition of 1-hexanol, and Leu30 also plays an important role in binding to this ligand.

The selective binding and transport of external volatiles by insects is accomplished through the synergistic action of various OBPs and CSPs (Liu et al., 2012). 1-hexanol is the main green leaf volatile in peach shoots and pear fruits (Piñero and Dorn, 2007; Najar-Rodriguez et al., 2013b). Electrophysiological experiments have shown that 1-hexanol can elicit strong EAG responses in male and female antennae of *G*. *molesta* (Li et al., 2016b). 1-hexanol is also a key component of a mixture (1-hexanol, nonanal, ethyl butanoate, butyl acetate, 3-methylbutyl acetate, ethyl hexanoate, and hexyl acetate, ratio 3:1:100:8:4:12:4) with attractive activity to adult *G*. *molesta* (Lu et al., 2014). Preliminary evidence from fluorescence competitive binding assays confirmed that GmolCSP8 participates in the recognition and transport of 1-hexanol. GmolCSP8 may serve as a target gene for *G*. *molesta* host-locating behavior.

## Author Contributions

G-WL, X-LC, W-QW, and J-XW conceived and designed the experiments. G-WL and X-LC performed the experiments. L-HC, G-WL, and W-QW analyzed and processed data. G-WL and J-XW wrote and edited the manuscript. All authors agreed to publish this manuscript.

## Conflict of Interest Statement

The authors declare that the research was conducted in the absence of any commercial or financial relationships that could be construed as a potential conflict of interest.

## Footnotes

1 http://www.cbs.dtu.dk/services/SignalP-4.0/

2 https://web.expasy.org/compute_pi/

3 https://www.ncbi.nlm.nih.gov/tools/primer-blast

## Supplementary Material

FIGURE S1 Melting curves and Standard curves of reference and target genes in qRT-PCR. **(A)** and **(B)** are the melting curves of Gmolβ-actin and *GmolCSP*8 genes, respectively. **(C,D)** represents the standard curves of Gmolβ-actin and *GmolCSP*8 genes, respectively.

FIGURE S2 Ramachandran plot of predicted of GmolCSP8 (KR003781) of *Grapholita molesta.*

FIGURE S3 Binding curves of 1-NPN to GmolCSP8 Wild-Type and its four mutants. rGmolCSP8. A 2 μM of each protein in 20 mM Tris–HCl buffer (pH 7.4) was titrated with aliquots of 1 mM 1-NPN stock solution to final concentrations of 0 μM to 24 μM, and the emission spectra were recorded between 370 and 550 nm. The calculated dissociation constants of rGmolCSP8 Wild-Type, T27A, L30A, V40A, and L80A were 13.85, 11.19, 14.38, 11.82, and 11.71 μM, respectively.

TABLE S1 Similarity and coverage of GmolCSP8 with other known crystal structure insect CSPs.

TABLE S2 Sequence identity analysis of GmolCSP8 and 14 other CSPs of *Grapholita molesta.*

DATA SHEET S1 Amino acid sequences of CSPs were used in phylogenetic analyses.
