# Supplementary figures and images for: Functional Analysis of the Chemosensory Protein GmolCSP8 From the Oriental Fruit Moth, Grapholita molesta (Busck) (Lepidoptera: Tortricidae)
Source: Front Physiol. 2019 May 7;10:552. doi: 10.3389/fphys.2019.00552 (PMC6516043; doi:10.3389/fphys.2019.00552)

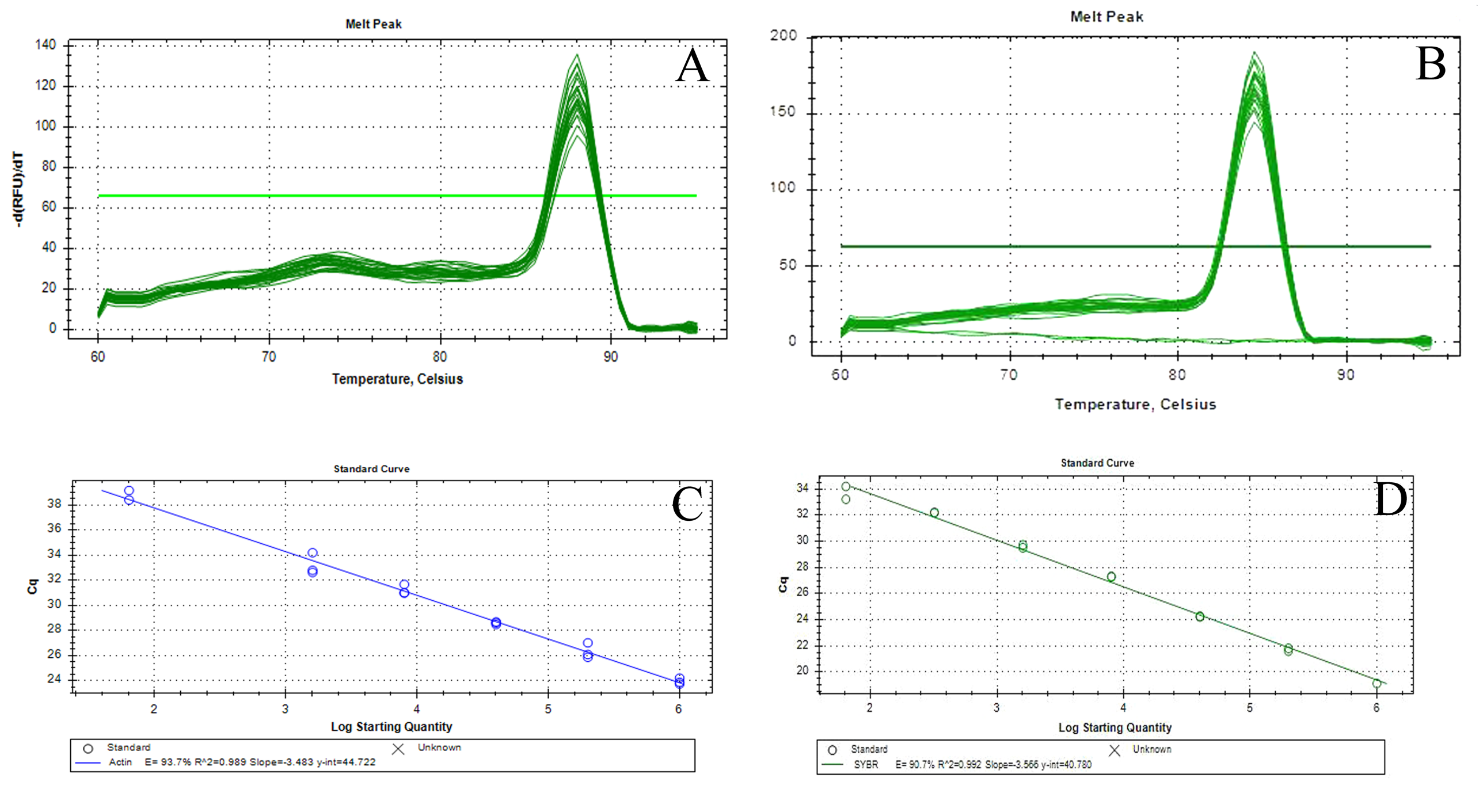

Supplement: FIGURE S1 — Melting curves and Standard curves of reference and target genes in qRT-PCR. (A) and (B) are the melting curves of Gmolβ-actin and GmolCSP8 genes, respectively. (C,D) represents the standard curves of Gmolβ-actin and GmolCSP8 genes, respectively. [file Image_1.TIF]

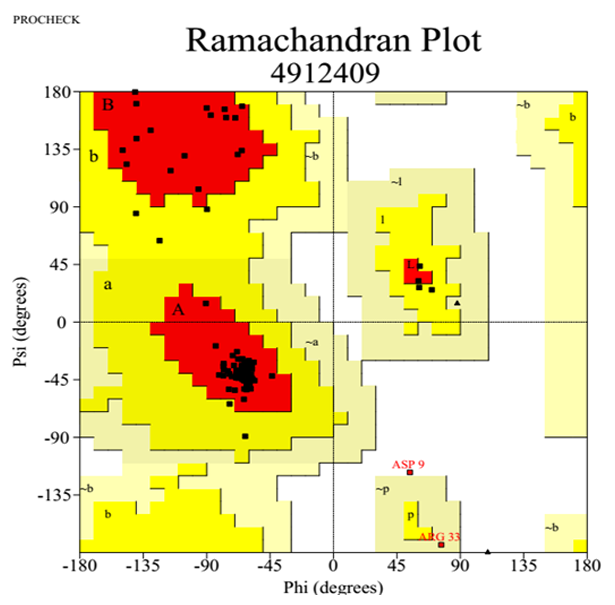

Supplement: FIGURE S2 — Ramachandran plot of predicted of GmolCSP8 (KR003781) of Grapholita molesta. [file Image_2.TIF]

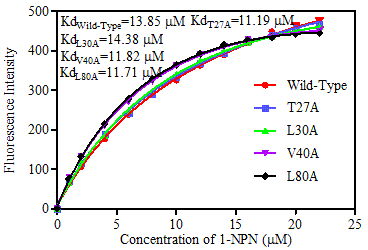

Supplement: FIGURE S3 — Binding curves of 1-NPN to GmolCSP8 Wild-Type and its four mutants. rGmolCSP8. A 2 μM of each protein in 20 mM Tris–HCl buffer (pH 7.4) was titrated with aliquots of 1 mM 1-NPN stock solution to final concentrations of 0 μM to 24 μM, and the emission spectra were recorded between 370 and 550 nm. The calculated dissociation constants of rGmolCSP8 Wild-Type, T27A, L30A, V40A, and L80A were 13.85, 11.19, 14.38, 11.82, and 11.71 μM, respectively. [file Image_3.PNG]
