# Supplementary material for: Functional Analysis of the Chemosensory Protein GmolCSP8 From the Oriental Fruit Moth, Grapholita molesta (Busck) (Lepidoptera: Tortricidae)
Source: Front Physiol. 2019 May 7;10:552. doi: 10.3389/fphys.2019.00552 (PMC6516043; doi:10.3389/fphys.2019.00552)
Supplement: TABLE S1 — Similarity and coverage of GmolCSP8 with other known crystal structure insect CSPs. [file Table_1.docx]

**TABLE S1** | **Similarity and coverage of GmolCSP8 with other known crystal structure insect CSPs**

| **Names of CSPs** | **Species** | **Identity (%)** | **Coverage (%)** | **PDB ID** |
| --- | --- | --- | --- | --- |
| CSPSg4 | *Schistocerca gregaria* | 46.46 | 95.0 | 2gvs.1.A |
| BmorCSP1 | *Bombyx mori* | 44.79 | 92.0 | 2jnt.1.A |
| MbraCSPA6 | *Mamestra brassicae* | 41.41 | 95.0 | 1kx9.1.A |
| MbraCSP2 | *Mamestra brassicae* | 39.81 | 99.0 | 1k19.1.A |
