# Supplementary material for: Functional Analysis of the Chemosensory Protein GmolCSP8 From the Oriental Fruit Moth, Grapholita molesta (Busck) (Lepidoptera: Tortricidae)
Source: Front Physiol. 2019 May 7;10:552. doi: 10.3389/fphys.2019.00552 (PMC6516043; doi:10.3389/fphys.2019.00552)
Supplement: TABLE S2 — Sequence identity analysis of GmolCSP8 and 14 other CSPs of Grapholita molesta. [file Table_2.docx]

**Table S2 |** **Sequence identity analysis of GmolCSP8 and 14 other CSPs of *Grapholita molesta***

|  | CSP1 | CSP2 | CSP3 | CSP4 | CSP5 | CSP6 | CSP7 | CSP8 | CSP9 | CSP10 | CSP11 | CSP12 | CSP13 | CSP14 | CSP15 |
| --- | --- | --- | --- | --- | --- | --- | --- | --- | --- | --- | --- | --- | --- | --- | --- |
| CSP1 | 100.00 | 23.64 | 34.78 | 31.93 | 16.83 | 34.19 | 28.57 | 37.39 | 31.09 | 30.77 | 31.62 | 16.81 | 30.17 | 33.91 | 34.45 |
| CSP2 |  | 100.00 | 18.26 | 19.82 | 19.59 | 21.62 | 18.58 | 23.89 | 20.35 | 24.35 | 22.02 | 15.45 | 18.75 | 19.13 | 21.24 |
| CSP3 |  |  | 100.00 | 36.13 | 26.00 | 29.66 | 46.72 | 43.70 | 41.73 | 37.90 | 33.90 | 23.01 | 48.33 | 69.92 | 38.66 |
| CSP4 |  |  |  | 100.00 | 21.36 | 41.18 | 36.29 | 45.30 | 30.65 | 27.27 | 47.54 | 20.00 | 38.84 | 44.54 | 48.36 |
| CSP5 |  |  |  |  | 100.00 | 20.20 | 22.33 | 24.49 | 25.24 | 20.59 | 21.78 | 15.69 | 26.00 | 28.00 | 18.45 |
| CSP6 |  |  |  |  |  | 100.00 | 28.33 | 32.48 | 25.83 | 24.17 | 38.46 | 20.54 | 30.25 | 32.20 | 43.70 |
| CSP7 |  |  |  |  |  |  | 100.00 | 44.54 | 40.16 | 44.35 | 34.96 | 23.08 | 47.15 | 47.93 | 31.45 |
| CSP8 |  |  |  |  |  |  |  | 100.00 | 35.29 | 40.34 | 43.48 | 23.21 | 42.37 | 45.38 | 42.86 |
| CSP9 |  |  |  |  |  |  |  |  | 100.00 | 37.90 | 35.77 | 23.93 | 40.65 | 42.15 | 33.06 |
| CSP10 |  |  |  |  |  |  |  |  |  | 100.00 | 35.00 | 26.96 | 36.07 | 35.77 | 33.06 |
| CSP11 |  |  |  |  |  |  |  |  |  |  | 100.00 | 26.55 | 36.13 | 35.04 | 49.17 |
| CSP12 |  |  |  |  |  |  |  |  |  |  |  | 100.00 | 26.32 | 24.78 | 22.22 |
| CSP13 |  |  |  |  |  |  |  |  |  |  |  |  | 100.00 | 79.17 | 33.88 |
| CSP14 |  |  |  |  |  |  |  |  |  |  |  |  |  | 100.00 | 37.82 |
| CSP15 |  |  |  |  |  |  |  |  |  |  |  |  |  |  | 100.00 |
