# Supplementary material for: Functional Analysis of the Chemosensory Protein GmolCSP8 From the Oriental Fruit Moth, Grapholita molesta (Busck) (Lepidoptera: Tortricidae)
Source: Front Physiol. 2019 May 7;10:552. doi: 10.3389/fphys.2019.00552 (PMC6516043; doi:10.3389/fphys.2019.00552)
Supplement: DATA SHEET S1 — Amino acid sequences of CSPs were used in phylogenetic analyses. [file Data_Sheet_1.docx]

>GmolCSP1 (MK640198)

MKFLIVLASLVLLAAGQDTYTPENDDLDIDALVSNPEALKAWFNCFVDKGQCDKVQTSFKDDLPEAIQQGCAKCTAAQKVILKKYLAGLKEKAPADYEVLRQKYDPENKYFGPLEKAIA

>GmolCSP2 (KR003779)

MRVLLLCMSCLLASVAAGDFEDLFKTDTTLLLTDESEWKVLFECLMERGPCGRYQDVKDKLLKLIGNKCEECTPEQKDLFYKTLKEFSNKYPKDYAMLNDKLLLSNVAQTTSSPS

>GmolCSP3 (KR003780)

MMLKYFVVLCVASWALADEKYTDKYDKIDLDEILGNKRLLQAYVNCILDKGKCTPEGKELKDHLEEALQTGCEKCTEAQKKGTSKVIDHLIKNELPIWRELSARFDPEGKFKKTYEQRAREHGITIPEE

>GmolCSP4 (MK640199)

MKVVLVTCVLLALAAARPEEGYSTKYDSFDAQELVDNVRLLKSYGNCFLDKGPCTAEGHDFKKTIPDALKTNCAKCSPKQRVLIRTVVKGFQSKLPELWKELVAKEDPNNEFHDDFNKFLNESD

>GmolCSP5 (MK640200)

MKLSLLVVAGVLACASAQARPPVTDTALEDALNDKRFIQRQLKCALGEAPCDPIGKRLKTLAPLVLRGACPQCTPQETKQIQRTLSYVQRNFPAEWAKIVRQYAG

>GmolCSP6 (MK640201)

MRVFLLFFLVYSVVSQEYYNRRYDYFDVETLVENPRLLQKYMECFLDRGPCTPVGRVFKRVLPELVATGCAKCSPSQRRFAKRTFEAFKRDLPESHAELKKKIDPTNKNYDNFERKIADA

>GmolCSP7 (MK640202)

MKTILIVCVLALAAVAYARPGDTYTDKFDNVNLDEILENRRLLIPYIKCVLEQGKCTPDGKELKSHIREALENNCAKCTETQRRGTRRVLGHLINHETDFWTQLCAKYDPDRKYVTKYESELRTVKA

>GmolCSP8 (KR003781)

MKTILIACLFAVAAAETKYDSSHDDIDLSEVLGNERLLVSYTKCLVDKGPCTPEVKQLKDKLPEALETNCAKCTDKQKTVGKQLVKELKEKHPDLWKQLVSKYDPNGKYHKAFEQFLRN

>GmolCSP9 (KR003782)

MKRLVFLVFLILLLCQSHAEDQTYTTKYDGIDLDEILASSRLLTGYVNCLLDLRPCTPDGKELKKNLPDAISNDCIKCTERQKQGADKVMHYIIDHRPDDWEKLEKKYDSDGSYKQKYLDSKEHKEEKATESEKPAENENAVETKTEISLEQQDK

>GmolCSP10 (MK640203)

MTLSIVIALCLFAVAVGRSQGKYTDKFDIDNIDEVLKSERLLKAYADCLMDRGRCSPDAKGLKVIVPEALENECYKCTEMEKQGAAKVIRNLVNEHPDIWKDLSSKYDPDNIYFRKYKNEFQIVIA

>GmolCSP11 (KR003783)

MLIASLALLSALSTVTADFYSSKYDDFDIQPLLANDRILLGYTKCFLDQGPCTPDAKDFKKVIPEALETICGKCTPKQKQLIRQVIRAIMDRHPESWKQLTGKFDPEDKHKDDFDKFLAENRK

>GmolCSP12 (MK640204)

MLSEIILSCLVTFRITSAEPTIAGIDRTMSDGVRSMGYNVMYGDEDFTVLNSVIDEVERKASKAKINLNELIQPLPAMDVKCLMSADHYCSKDMKETKSVIIQAIKDDCKDCSTPQKENAGRVIAAMMAHDPTSWKLFLTRYDGIKKIQRIMG

>GmolCSP13 (MK640205)

MKLLVLCLGLVCLAIAEDNKYTDRYDNINIDEILANKRLLGPYVKCLLGQGRCTPEGKELKNTIRDAMTTSCEKCTEQQRKGARKVVKHLKEKEPEYWNQIKAKYDPGDKFQESYEAFLARDD

>GmolCSP14 (MK640206)

MMLKYFVVLCVASWALADEKYTDKYDKIDLDEILGNKRLLQAYVNCILDKGKCTPEGKELKDTIRDAMTTSCEKCTEQQRKGARKVVKHLKEKEPEYWNQIKAKYDPGDKFQESYEAFLARDD

>GmolCSP15 (MK640207)

MRIEMKSCIVFTCLLAAVLAADKYNSKYDNFDVETLITNDRLLKSYINCFLDKGRCTPEGTDFKKTLPEAVETQCAKCTEKQKGNIKKVIKAIQTRHPRQWDDLVKKNDPTGKHIANFNKFIDS

>BmorCSP1 (XP_021205890)

MKCLTIAALLFVAGLSIAEKYTDKYDNIDVDEILENRKLLVPYIKCVLDEGRCTPDGKELKAHIKDGMQTACAKCTDKQKVSARKIVKHIKQHEADYWEQMKAKYDPKDEFKEIYEGFLAGQN

>BmorCSP2 (NM_001098308)

MKSVILICFLGVATVVIARPKTPFDNINIEEIFENRRLLLGYINCILERGNCTRAGKDLKSSLKNVLEENCDKCSEDQRKSIIKVINYLVSSEPESWNQLKSKYDPEGKYLIKYEAKMESN

>BmorCSP3 (NM_001043598)

MNSLIAFCLFAVLAVALARPDDKYTDRYDNVNLDEVLSNSRLLQPYIKCILDKDRCAPDAKELKEHIREALETECAKCTEAQKKGTRRVIGHLINNESKSWNELTAKYDPENKFTAKYEKELREIKA

>BmorCSP4 (NP_001037052)

MKVLIVLSCVLVAVLADDKYTDKYDKINLQEILENKRLLESYMDCVLGKGKCTPEGKELKDHLQEALETGCEKCTEAQEKGAETSIDYLIKNELEIWKELTAHFDPDGKWRKKYEDRAKAKGIVIPE

>BmorCSP5 (NM_001043597)

MKTVIVCLLALTAVALARPEQYTDKYDTVDLDQLISNRRLLIPYVHCILEKGQCTAEGKELKSHIKEALETNCAKCTKAQKGGTEKMIGHLINHEAEFWEELKAKYDPTNEFTKKYETELKRVTA

>BmorCSP6 (XP_012549349)

MKSLIVLSCLLAACLAADLSKYENFDVEPIVTSDRLLKAYINCFLDKGRCTPEASDFKKALPDTIATNCGKCTEKQKANVRKVIKVIQQKHSTEWEKLVKKHDPSGKHRADFDKFLLGS

>BmorCSP7 (NM_001043603)

MKGFYVLCFALFAAVYCKETYSSENDDLDIEALVGNIDSLKAFIGCFLETSPCDAVSGDFKKDIPEAVAEACGKCTPAQKHLFKRFLEVVKDKLPQEYEAFKTKYDPQGKHFDALLSAVANS

>BmorCSP8 (NM_001043602)

MKTILILCALVSVVVCRPEEYYSSQYDNFDVEQLVGNLRLLKNYAKCFLDQGPCTAEGTEFKKRIPEALRTKCAKCNPKQRHLIRTVVKAFQTKLPDLWEELAIKEDPKGQYKHEFTAFINAMD

>BmorCSP9 (NM_001043604)

MRAVIFLYTCVFVVVGQDINAMMSMPKYDERYDYLDVDDIFRNKRLVRNYVDCLINAQRCTPEGKALKRILPEALRTKCIRCTERQKRTSVKVIRRLKNEYPEEWAKLASRWDPTGDFTRYFEDYLAKEHFNTIPGSGPTVNVLSLQTTPPPPPPPPSRPASVFTNPPPPVMSTSPRPVVLNRFRR

>BmorCSP10 (XM_012693783)

MKILIIVVMACVAVTWARPESTYTDKWDNINVDEILESNRLLKGYVDCLLGKGRCTPDGKALKETLPDALEHECVKCTGKQKSGADKVIRHLVNKRPDLWKELAVKYDPDNIYQARYKDKID

>BmorCSP11 (NM_001098309)

MKLTSFLLVGMAMVSAEFYSSRYDDFDVKPLVENDRILQSYTNCFLDKGPCTPDAKEFKKVIPEALETTCGKCSPKQKQLIKTVIKAVIERHPEAWEELVNKYDKDRKFRPSFDKFINEDD

>BmorCSP12 (NM_001098310)

MFMLFIISFIIVPVLKCCGTETSTYTTQYDEVDIKEIMGNERLLVAYIGCLLDKNPCTPEGKELKRNIPDALQSDCSKCSDKQRENADAWIEFMIDNRPEDWTKLEER

>BmorCSP13 (NM_001043715)

MKLLLVFLGLFLAVLAQDKYEPIDDSFDASEVLSNERLLKSYTKCLLNQGPCTAELKKIKDKIPEALETHCAKCTDKQKQMAKQLAQGIKKTHPELWDEFITFYDPQGKYQTSFKDFLES

>BmorCSP14 (NM_001043727)

MKSSLFCVLVLTVVVSSSRQQSYPRNDNININAILQNDRILLGYFKCVMDRGPCTKDGKTFKRALPEALPTACARCSNKQKAAFRTLLLAIRARSEPSFLELLDKYDPSRSNRELLYTFLATGL

>BmorCSP15 (XM_021350360)

MIENFYSKCTISKSVLFLCLIFLPYALNQKYYDSRYDYYDIDHLVQNPRLLKKYLDCFLGKGPCTPIGRLFKQVMPEVITTACAKCTPTQKRFARKTFNAFRRYFPETLMELRRKFDPESKYYDAFEKVITNA

>BmorCSP16 (NM_001098312)

MIEWKRFKILHFLSYLGLLVLVVVCAAQQNRPQVTDTALDEALNDKRFIQRQLKCALGEAPCDPIGKRLKTLAPLVLRGACPQCSPQETKQIQKTLSYVQRNFPQHWAKLVRQYAG

>TcasCSP1(NP_001039273)

MLILQIAHLCAQFCLLAAIFTCVKPQLTRISDEAIESTLNDRRYLLRQLKCATGEAPCDPVGRRLKSLAPLVLRGSCPQCTPQEMKQIQKVLAFVQKNYPKEWNKILHQYAG

>TcasCSP2 (NP_001039277)

MKIIILAVLIATAVAATYDVYPTKYDNVDIDAILHNKRLFDNYLQCLLKKGKCNEEAAILRDVIPDALITGCRKCNDHQKVSVEKVIRFLIKERNSDWQQLISVYDPKGEYQTQYAHYLEKI

>TcasCSP4 (NP_001039285)

MYSYLIPLYLFLFVHYGWSEDTTHKYTTKYDNIDLENVVKNERLLKSYVDCLLEKGRCSPDGLELKKNMPDAIETDCSKCSEKQKEGSDFIMRYLIDNKPDYWKALEAKYDPDGTYKKRYFESQKDEVSKVEA

>TcasCSP5 (NP_001039287)

MKTFVILFFGVFFIIFSDFVNGKTLHRSTRDDKYTTRYDNVDVDRILHSKRLLLNYINCLLEKGPCSPEGRELKKILPDALVTNCSKCSEVQKKQAGKILTFVLLNYRNEWNQLVAKYDPDGIYRKQYEIDDDYDYSELDSAKK

>TcasCSP6 (NP_001039288)

MIPLIAIAGILAVSAAPAEFYESRYDHLDVESILNNRRMVNYYAACLLSKGPCPPQGVDLKRVLPEALQTNCAKCTEKQRTAAYRSIKRLKKEYPKIWEQLRAVWDPDDVFIRKFETSFESGKPSGVISTNTSPPSPILSNRFGENEEADAASNVISSTPLPPTTSTTTRTTLTTKFTTKPSTKPTNKPVVVTKPPQAPPFATVGANLQATVSFGTNLVGGIVRSLGTLGSRVVESGTKLANMVISAAIRP

>TcasCSP7 (NP_001039289)

MKLISAVILCAFLVAVSAAENKYTNKYDNVDVDKILNNDRVLTNYIKCLMDEGPCTSEGRELKKTLPDALSSGCTKCNQKQKETAEKVIRHLTQKRARDWERLSKKYDPQGQYKKRYEEHVATSRAA

>TcasCSP8 (NP_001039290)

MPLVKSLVVVVLLIGVVYQVQGQLGLAGNNYIEKQLLCALDKAPCDALGNQIKGALPEIIGKNCERCDSRQVANARRIARYVQTKHPDVWNALVKKYSV

>TcasCSP9 (NP_00103928)

MTAIVFLLALACLKTYVSSQEYLVPQNIDVDEILKNDRLTRNYLDCVLGKGKCTPEGEELKKDIPEALQNGCAKCNEKHKEGVRKVIHHLIENKPNWWQELESKFDPQGEYKKKYDELLKKEGLAN

>TcasCSP10 (NP_001039278)

MKTFVLVAFAAVLGLALARPQEKYTTKYDNIDLEEILKSDRLLKNYFNCLMERGTCSPDGEELKKALPDALHSGCSKCTEKQKEGSRKIIHYLIDNKRDWWNELEAKYDKDGVYRQKYKDVIEKEGIKL

>TcasCSP11 (NP_001039279)

MKTLVPLLFFVIAIASSLAENSKYTTKYDNVDLDEIIKSDRLLKNYVNCLLEKGKCTPDGAELKRHLPDALHTECSKCSETQKNGSKKIMRHLIDHKRDWWNELEEKYDKEGEYRKKYEAEIKGKKD

>TcasCSP12 (NP_001039280)

MKTLVLVLFVAVLSVVFAADKYTTKYDNIDLNQILKSDRLLKNYVNCLLDRGKCSPDGQELKNNLADALQTSCSKCSQRQKDGSRTIIRYLIKNKRDWWNELEAKYDPTGIYKNKYADELKAEGIVL

>TcasCSP13 (NP_001039281)

MFLAIVLVVCACTNVLSEEYTNQYNDELDAALKSERLMKSYFECLLGTGKCTPSGEELKKDIPDALKNECAKCNDKHKEGIRKVIHYLVKQKPEWWEQLQKKFDPQGIYKKRYQNYLDKEGLKA

>TcasCSP14 (NP_001039282)

MFATSALFAFICIQGLVSAEEYLVPQNIDLDEILKNDRLTRNYIDCILGKGKCTPEGEELKRDIPEALQNECAKCNEKHKEGVRKVLHHLIKNKPNWWQELEAKFDPKGEYKQKYNKLLEKEGLQA

>TcasCSP15 (NP_001039291)

MIFKIHFLVFGALLTYVSSVEYLILREIDTILKNDQMTRNYLDCVLDKGKCTKEAEKLKKGITETMKNGCVKCEQKQKEDVHKVFQHLMIHRPNWWHELETKFNPHHEIKLQHLHQSKFNPHEEVKLQHLHQFPHHDFLEREGFIR

>TcasCSP17 (NP_001039284)

MFKVLFVVFACVQAYVYAEEYTVPQNIDIDEILKNDRLTKNYLDCILEKGKCTPEGEELKKDIPDALQNECAKCNEKHKEGVRKVIRHLIKNKPSWWQELQEKYDPKGEYKSRYNHFLEEEGLN

>TcasCSP18 (NP_001039286)

MLFTVFLVLTCAHVVFLEEYVIPDNIDIDDILSNERLLKNYVNCLLDKGRCTPEGKKLKSTIPEALSTDCAKCNEKVKANVRKVLHHLIDNKPDMWKQLEAKYDPSGEYRSKYKDELEKNGIHV

>TcasCSP19 (EFA07577)

MKFFIAFLMLLGAVWCEQYTTKYDNINVDEILASERLLKNYFNCIMDRGACTPDADELKRVLPDALKSDCAKCSEKQKEMTKKVIHFLSHNKQQMWKELTAKYDPDGIYFEKYKDKFDS

>TcasCSP20 (NP_001039274)

MRFFVIFFVACVSVALARPEDQYTIKYDNVNLKEILQSDRLTENYVNCLLEKKPCTPDGEELKRVLPDALKTSCAKCTDKQKQGAKTVIQHLYKNKQDWWKQLEAKYDPEHTYVKAHEDELKAL

>AaegCSP1 (EAT46822)

MNTTKLVMLSATLIVALMVFNWPQPAAANDSQNLNRLLNNQVIVSRQIMCVLEKSPCDQLGRQLKGRSGVDF

>AaegCSP2 (EAT46827)

MKSVCLIVFGVVALVATVSAQQKYTDKFDNINVDQVLSNDRILSNYLKCLLEKGPCTQEGRELKKTLPDALRTNCEKCSEKQRTNSRKVISHLESKKPAEWKKLLDKYDPEGIYKSKFEKLNKRS

>AaegCSP3 (EAT46830)

MERKSTGCNPPQSSPPVLQTRLNRRAPSGEIPSDWDLEWQLFVPRIGIYPRTGKRGIVRCEKKIEREAKKPEENAVLKLNQCANKSGRYECELSSEQKKCFVRVASALLAFANFVKSQDSARNLYSSRYDNLDIDTILGSNRLVNNYVDCLLSRKPCPPEGKDLKRILPEALRTKCARCSVTQKENALKIITTLYYSYPDQYMALRERWDPSGEYHRRFEEYLQGIQFNQIGSNGNDRPVRNDFDRDQSQVLLQTLILSSTTVAPSSPPQPQPQSPNALPNGPEQRPQQLAPSAQLTQPAEATKKLE

>AaegCSP4 (EAT46831)

MSHKFCWIVVICAISIINVNCYDTKYDNVNLDEIFKSTRLLNNYINCLKNMGPCTPDAKELKELLPDALESECAHCTEKQKVGAERVINFVVDNRPDDFKILESMYDPAGEYRRKYLRDHPNFHDQGAPLTAADATENPPSSNGGDEAPTEESQQNQGQSEDGDDRRR

>AaegCSP5 (EAT46832)

MKLFAVVALALFAVAAAQEKYTTKYDGVDLDEILKSDRLFNNYYKCLMDQGRCTPDGNELKRVLPDALKTDCAKCSPKQRDGTQKVVNYLIDNRPSQWKNLQAKYDPQNIYVEKYRTEAKKAGIKL

>AaegCSP6 (EAT46834)

MKYFFVVFLALAATVIAQNEINQYTTKFDSIDVDEILKSDRLFNNYYKCLLDLGRCTPEGVELKRVLPEALETSCAKCSEKQRETSDRAIKYLTENRPEEWKVLKARYDPDNKYSKKNENDA

>AaegCSP7 (EAT46835)

MKLFIALALLAVAAAQEATYNNRYDNIDVEEILKSDRLFKNYFNCLMDAGPCTPEGTDLKKYLPDALETGCTKCTEKQRDTGNKVIAWLIENRPMEWVMLKSKYDPENKLTERYRELAAKAGIAL

>AaegCSP8 (EAT46836)

MKIIILCTLLAVVAAQEATYNNRYDNIDVEEILKSDRLFKNYFNCLMDAGPCTPEGTDLKKYLPDALETGCTKCTEKQRDTGNKVIAWLIENRPMEWTMLKNKYDPENKLTERYRELAAKAGIAL

>AaegCSP9 (EAT46837)

MKFLVAVLSLIAVAAAQDLYTTKFDNIDVDEILKSDRLFKSYYQCLLDEGRCTPEGNELKRSLPDALETGCSKCSEKQRSAGVRAVKYLSENRPTEFKALRNRFDPENKYVEQYVRDAEKEGITLNI

>AaegCSP10 (EAT46838)

MKIFIAVFTLMAVVAAQEFYTSKFDNIDVDEILKSDRLFKNYYQCLLDQGRCTPEGNELKRVLPDALETACSKCSEKQRSAGVRAVKYLSENRPAEFKALRARFDPENKYVDQYVRDAEKEGITLNIS

>AaegCSP11 (EAT46839)

MKFFVVALALIAAVAAQDEAMYTSKFDNINLDEILQSDRLFKNYYNCLTDAGPCTPEGNELKRVLPEALETNCAKCSPKQREAGTRAIKHVTENRPEEWKVLRARFDPENKYIERFSAEAEKEGIKL

>AaegCSP12 (EAT46840)

MDHSSISASKPSAHFKVLNRSFTHKFGATDTIMKIFVVALALIAAVAAQDEAMYTSKFDNINLDEILMSDRLFKNYYNCLTDAGPCTPEGNELKRVLPEALETNCAKCSPKQREAGTRAIKYVTENRAEEWKVLRARFDPEDKYVAQYLAEAEKEGIKL

>AaegCSP13 (EAT46841)

MKFFIVVLALFAVAAARPQEDKYTTKYDSIDIDEILKSDRLFKNYFNCLMDTGACTPEGNELKRVLPDSLENNCSKCSEKQQTSSTKIIKFLTENKPEEWTMLKAKYDPDNKYVQKYVADADKDGIKL

>AaegCSP14 (EAT46842)

MKFFIVALALFAAAAARPQEDKYTTKYDSIDIDEILKSDRLFKNYFNCLMDTGACTPEGNELKRVLPDSLENNCSKCSEKQQTSSTKIIKFLTENKPEEWTMLKAKYDPDNKYVQKYVADADKDGIKL

>AaegCSP15 (EAT46843)

MKFSIVVLALFAVAAAKPQDDKYTTKYDSIDIDEILKSDRLFKNYFNCLMDTGACTPEGNELKRVLPDSLENNCSKCSEKQQTSSTKIIKFLTENKPEEWTMLKAKYDPDNKYVQKYVADADKDGIKL

>AaegCSP16 (EAT46844)

MKFFIVVLALFAVAAARPQDDKYTTKYDSIDIDEILKSDRLFKNYFNCLMDTGACTPEGNELKRVLPDSLENNCSKCSEKQQTSSTKIIKFLTENKPEEWTMLKAKYDPDNKYVQKYVADADKDGIKL

>AaegCSP17 (EAT46845)

MKFFIVALVLIAVAAARPQDDKYTTKYDSIDIDEILKSDRLFKNYFNCLMDTGACTPEGNELKRVLPDSLENNCSKCSEKQQTSSTKIIKFLTENKPEEWTMLKAKYDPDNKYVQKYVADADKDGIKL

>AaegCSP18 (EAT46846)

MKFFIVALVLIAVAAARPQEDKYTTKYDSIDIDEILKSDRLFKNYFNCLMDTGACTPEGNELKRVLPDSLENNCSKCSEKQQTSSTKIIKFLTENKPEEWTMLKAKYDPDNKYVQKYVADADKDGIKL

>AaegCSP19 (EAT46847)

MKFFIVVLALFAVAAARPQEDKYTTKYDSIDIDEILKSDRLFKNYFNCLMDTGACTPEGNELKRVLPDSLENNCSKCSEKQQTSSTKIIKFLTENKPEEWTMLKAKYDPDNKYVQKYVADADKDGIKL

>AaegCSP20 (EAT46848)

MKFFIVALALIAVVAAQDDKYTTKYDSIDIDEILKSDRLFKNYFNCLMDTGACTPEGNELKRVLPDALENNCSKCSEKQQTSSTKIIKFLTENKPEAWTMLKAKYDPDNKYVAKYVADADKEGIKL

>AaegCSP21 (EAT46849)

MKFFIVALALIAVAAARPQDDKYTTKYDSIDIDEILKSDRLFKNYFNCLMDTGACTPEGNELKRVLPDALENNCSKCSEKQQTSSTKIIKFLTENKPEEWTMLKAKYDPDNKYVAKYVADADKKGIKL

>AaegCSP22 (EAT46850)

MKFFIVALALIAVAAAQDDKYTTKYDSIDIDEILKSDRLFKNYFNCLMDTGACTPEGNELKRVLPDALENNCSKCSEKQQTSSTKIIKFLTENKPEEWTMLKAKYDPDNKYVQKYVAEADKDGIKL

>AaegCSP23 (EAT46851)

MKFFIVALALFAVAAAQDDKYTTKYDSIDIDEILKSDRLFKNYFNCLMDTGACTPEGNELKRVLPDSLENNCSKCSEKQQTSSTKIIKFLTENKPEEWTMLKAKYDPDNKYVQKYVADADKDGIKL

>AaegCSP24 (EAT46852)

MKFFIVALALLAVVAAQDDKYTTKYDSVDIDEILKSERLFKNYYACLMDTGACTPDVNELKRVLPDALENNCAKCSEKQQNDSTKTIKYLTENKPEEWKALKAKYDPDNKYVEKYVADADKEGIKL

>AaegCSP25 (EAT46853)

MKIFIVALALIAVVAAQDDKYTTKYDSVDIDEILKSERLFKNYYACLMDTGACTPDVNELKRVLPDALENNCAKCSEKQQNDSTKTIKYLTENKPEEWKALKAKYDPDNKYVEKYVADADKEGIKL

>AaegCSP26 (EAT46854)

MKFFIFALALIALAAAKPQDDKYTTKYDSVDIDEILKSERLFKNYYACLMDTGACTPDVNELKRVLPDALENNCAKCSEKQQNDSTKTIKYLTENKPEEWKALKAKYDPDNKYVEKYVADADKEGIKL

>AaegCSP27 (EAT46855)

MKFFIVALALIALAAAKPQDDKYTTKYDSVDIDEILKSERLFKNYYACLMDTGACTPDVNELKRVLPDALENNCAKCSEKQQNDSTKTIKYLTENKPEEWKALKAKYDPDNKYVEKYVADADKEGIKL

>AaegCSP28 (EAT46856)

MKIFIVALALIALAAAKPQDDKYTTKYDSVDIDEILKSERLFKNYYACLMDTGACTPDVNELKRVLPEALENNCAKCSEKQQNDSTKTIKFLTENKPEEWKALKAKYDPDNKYVEKYVADADKEGIKL

>AaegCSP29 (EAT46857)

MKIFIVALALIALAAAKPQDDKYTTKYDSVDIDEILKSERLFKNYYACLMDTGACTPDVNELKRVLPEALENNCAKCSEKQQNDSTKTIKFLTENKPEEWKALKAKYDPDNKYVEKYVADADKEGIKL

>AaegCSP30 (EAT46858)

MKLFIVALALLAVVAAQDDKYTTKYDSVDIDEILKSERLFKNYYACLMDTGACTPDVNELKRVLPDALENNCAKCSEKQQNDSTKTIKYLTENKPEEWKALKAKYDPDNKYVEKYVADADKEGIKL

>AaegCSP31 (EAT46859)

MKIFIVALALIALAAAKPQDDKYTTKYDSVDIDEILKSERLFKNYYACLMDTGACTPDVNELKRVLPDALENNCAKCSEKQQNDSTKTIKYLTENKPEEWKALKAKYDPDNKYVEKYVADADKEGIKL

>AaegCSP32 (EAT46860)

MKIFILCAIMAVVAAQEATYNNRYDNIDVEEILKSDRLFKNYFNCLMDAGPCTPEGTDLKKYLPDALETGCTKCTEKQRDTGNKVIAWLIENRPMEWVMLKSKYDPENKLTERYRELAAKAGIAL

>AaegCSP33 (EAT46861)

MKLFIALALLAVAAAQEATYNSRYDNIDVEEILKSDRLFKNYFNCLMDAGPCTPEGTDLKKYLPDALETGCTKCTEKQRDTGNKVIAWLIENRPMEWVMLKSKYDPENKLTERYRELAAKAGIAL

>AaegCSP34 (EAT46862)

MKLFVAVFALIAVVAAQELYTSKFDNIDVDEILKSDRLFKNYYQCLMDEGRCTPEGNELKKILPEALETNCAKCSEKQRDGAIKAFGYLSENRPTEWKTLRDRFDPEGKYIEQYREEAEKNGIKF

>AaegCSP35 (EAT46863)

MKIMIVIAFALLAVASAQEEQYTTKYDNIDVEEILKSDRLFNNYFKCLMDEGPCTPDGNELKRILPEALQTNCAKCSESQRAGAIKVINYMIENRAEQWKALQEKYDPENIYLEQYRAEAEQSGITL

>AaegCSP36 (EAT46864)

MKSFIVIALALVVAVAAQNKYTSKYDGVDIDEILKSDRLFNNYYKCLLDQGRCTPDANELKRILPEALKTNCEKCSEKQREGATRVINYLIENRNQQWQTLQAKFDPENIYINQYRNEARAAGIKI

>AaegCSP37 (EAT48212)

MNSVNRYVLCIALIALFVASFTTAEENCEISANELGKIEQTLTHINQPIYTGDDESEVSDSDQCAQMLRGIHFQLRRLTQKYKLMNKGYVKAEEFAKMARDYEDQLSVLKNDLEQLKIGADSSAKQKMQELKKDIATLEQNVNTLHKDLEGITDELGKVRMDLCLTYMESNQLSNAQDKVKTLAPKYLMELVEQFLNKSEKNWLPVVDLS

VAIPDLDDRGQVYKTVHEFLKTKNRDGGEDSILLEAEVLKMNATFHPGSKITEDRKKEIQDLLEKLSLTSTKIFDQWTQDLAKLENSAVYKNSIDRMFLTQMEKFGERVMAKDDYYSLRNFLKLLVVSTNYYKIAAYRKLIQEKIGHTLAVLMFDMMSMERTELQYDPHVPDEVVRMYDESITALPDSLKNIRSCLKLVKIYNHVTNQCILATNEVEDVNNSNPKFKSNVLGRRKLVKTASNDCTPFRLEPSADKASIRIITPKGDALTNINSIQPGLSWFNRVGAPYTNNHNMKLDYSADWILDANYANDSIKIESEFNAYQTMKSVDHLMVTNVGKVPHVVVAQYGLKGMEYAGAGMKDAEWKFKCDN

>AaegCSP38 (EAT48213)

MKLKVYICQVIFSFLAVSVFCEENCNIPESELSKIDHVLRHMEKPIYSEEQFASDNEECTNLLNGIHAQLRRLTQRYKLMNKGYVKVEEYQRMADDYEKQLKTLNDELVELQQHTSEKASATIAKLKEDIKKLDEEVGTLHEKLKGIKQDFEKVKRDLCVTYLNSNQMSKAKAKLKEMASTYLIEIVQQQLNKSNANIMPMLEFSAAIPDLDDMGEAYKEIYKFLEEQKRLEGEDSVLLEATVLKMNASLKEGSNITDERRTQIEGLLKDLATKSTIVFSTWTKELKKINDAVVIKNALDHMFVSQMKVFGALVGDTSDFGSIRNFVKLTVVCNNYYKVAAYKELIDRKIGNALGTIMFDLLTLEVNEMKFDPHVPDEIPKLFEATLSSLPNSLTELRTCLGKVQIYNKKTNKCVVATGNDFDVHKDKLGDFYRVVVADYGCTSFRLEASGDKASVRIVTPSGNPMSNVNLHLEGNSLHNYVATPKSNKPDRTPSSSDEWILDANYNNDTIKIESQFSDYKTKKTEVDHLLVRDINHLPHVLVARYGFMGLKNSDAKDTIEWNLKCGS

>AaegCSP39 (EAT48214)

MARGMGCWQPASQWGHKGFGFVTFQSEDVVDKVCEIHFHEINNKMVECKKAQPKEVMLPANLAKTRTAGRGTYDFMWSLGTLPDGFPAAAYAAYAAGRGFSGYPSFGLPYPTGNLNLAALHAHLAAAAATASAGGPHHHHHAHSSSSGNNTTPPPPTRSNPALVQLEATRIDCCLIPGGEHRTTYNSTNMHEMHFNPTSCRMRIVSLVGLVGSDRAYHPPPGTGAGESKLDKGIRHRSSIHSGAYTQLLCIRPNVCEGNYTRVDVAGEFVSTFPARFLDHYIVIVVVRRRSKALSSFDSTPPSTL

>AaegCSP40（EAT48215）

MNRQLWIIIFAILCVAQAEEDNPTTEKMEELGIATINNFTREFYSYVEAVSQVLADLELTTTASITQIKHRIKHLLQEKCNLCSAKAEGPALDQGYVTTSNGSVIPVSYEQTRFGGGWIVLMQRYDGTVRFNRSWAEYRDGFGMVGHEFWLGLERIHQMTKDAEYELMIEMQDFEGNYKYAGYDAFAVGPEEERYPLAKVGKFNKTAYVDSFGKHRGYGFSTYDNDDNGCSNQYGRGGWWYYRKSCFGASLTGIWQNKQDWKSISWVWFSTEKKQVPLKFARMMMRLKTAE

>AaegCSP41（EAT48216）

MILQFWVVTFSVLFAARADENHSILIKLNDLDHRFTQMFSQQFYRHTQQVTDRVSALKISIDTNLLELDQQIQQALDGIQSNESSSSASATKPPGLTTIPIGSEPRVPALYERERYGGDWLVVMHRYDGSVKFDRTWAEYRDGFGMVGQEFWYGLERLHQLTKEKSYELMVEMEDFNGSLKYAWYDKFVVGPEEQRYALVELGTFNGTTDGDSLKPHKGSGFSTYDNDDFGCSNKYAKGGWWYYSGKCYGSSLTGIWKNELAYSSIVWMKFSDVSNTPLKLVRMMIRPKN

>AaegCSP42（EAT48217）

MVIQRVWRSSANSSTSGNTTATNSNSGALTASSLSATSSSELAPQNVPSGEIDGSQVVVPKMQRPKLSGEAMLEVMKMRYSGRSHSSQSAGDAALKSLELLRANIQYLFDKEIEVVVKKFSSLFFIPAIKNIKENLGESAISDDTLKTLYCSLLENSKSQYVGQIASPAESSLSRANTPGMELSDSDSSNDNVVPSGTTSLLQQALKRKLPEPNQHDGFKRQYFLQGSLYSQNHYSILQSLGNVQGSLPYQIRPSVLNPTVYTTTISPETLFIMDFKAGRALGVPDFRDRLANKHPEILRYCPDNQDRDWLLQQKQISPLNRNGRFFLLVLDEVRKLAERNSEYSNNPYMKLSDLQGFKLTEFIYAKVQKLIKDSADSSVKPTTATPSVTTATTAVPNSIQPRPRVSSLSSSHATLTALLSSPQQSQVNCSNSSGTIATIAGSSTTSGMDANTGGTGSGDTKT

>AaegCSP43（EAT35447）

MFSKVKSLSTRGAASTCTVRLVLVVVFMLAISQVAAQSSSTTTPEATSSSGNNGSSPSTPNKSQVSDEALDKALNDKRYLMRQLKCALGEVPCDPVGKRLKSLAPFVLRGACPQCTPAELVQIKKTLAHLQRNFPAEWNKLVQTYAG

>LstrCSP1 (AGZ04929)

MASVSSATLTAAALLALLALQLTAAQNFNEADIARMLNDSGLVQRQISCILGEAACDNIGNMLKLAIPEVLKRNCRSCNAQQASNARRLISFVQANYPAQWQRIQSRYVG

>LstrCSP2 (AGZ04930)

MRCLLLVAVICAAFIAAAQADEANKYTSKYDNIDIDKILKNDRVLSQYIKCLMGEGSCTQEGRELKRLLPDAIQSNCSKCSEKQRQASVKVMRHLRQSRVRDWNRLLDKYDPQGDKRKNLKLD

>LstrCSP3 (AGZ04931)

MKLALFCCLLGLVIAVSAEKYTTKYDHINVEEILNNERLFNSYYKCLMGGKCTPDGLELRTHLPDALRTNCSKCSEKQKEFSDKVIRYLIDNKPEEFAALTKKYDPEGIYKTTFGPQFKKDNTTTNQ

>LstrCSP4 (AGZ04932)

MLWAAKFIVFPLIFCVLQVWSAPADEKYSDIDFESILANRRVLSSYVKCLTDKGPCTPQGKELKKIVPEVIQTSCTKCSPQQKKVVRNVITTMQSKYKDQWDLVVNKYDPKKQRAGELKAFLAGTD

>LstrCSP5 (AGZ04933)

MLKFKLTLLVMASAFFSVDGGKLYKDRYTTKFDKIDLDEALNNQRLFESYLKCLMGDKCSPDGYELREALPDALATACAKCSEAQKAGTEKVIRFLIEKRPKEYALLEKKYDPEGVYRDKYKPIAEEKGIKI

>LstrCSP6 (AGZ04934)

MQASSLAMLLIAVWVLSPRRPLSGGFAGVHAQQSKNTRYTTRFDSIDVEVILKNERIFKRYMDCLLDKGRCTPEARELKRLLPEALKTECLKCSEVQRRQGAKVMAFIIKNKRPYWDLLLAKYDPQGVFRAKYKYNDQNIEAVLKQLEREQQGLYGTYSNPTNTTTVNSASSRK

>LstrCSP7 (AGZ04935)

MIQRTQGFNSIVVLLLIKLTVLSMVLASTHAPAPTPTPKVETKATEAAKSSSKDEIPDQTFDRYINNERYMLQQYECLMGNKPCDHVGRKLKAAVPLVVRGLGCPKCSPREEEQMKRIVSHVQRSYPDKWQKLIRKYGQ

>LstrCSP8 (AGZ04936)

MKSPCLLSVSCCILVLVASSASAAPKEKDPERKALYRLEYIDIEKVLDNNRMLTNFIRCFLRKGPCSPEARDFRKLLPKLAKTMCSDCSPRQRFIIKKVFKHLMEERPKEWELLMDRFDPQRKYAERLDTFMVDMTTRATPTTTTTTIPTTTTPMSSTTQRIIEILRTSTEMSNESRP

>LstrCSP9 (AGZ04937)

MSEVLVMILIFMLLAGREQRLQQQQQQQQQPQPQQQNVDNIEMSIYDKMFENMDVNSLLKNHRLVDSYLKCFLNEGSCTHIGHEVKMMIPEVIRSKCATCGENQMRALKAGLRLFIALRPDDWKRFLDVYDPDRTEWPHIKAFMEYDD

>LstrCSP10 (AGZ04938)

MYDHINVYNILKNERLFNRYFTCLTKKEGCTPEGKLLAAAILDALETSCANCSNEQRKLAEQVIQYLYFNKRDKFDELAMIYDLEGVFQEYHIAEYLVSGSWMPDFRKLPPV

>LstrCSP11 (AGZ04939)

MKFLYFTVFGCALVMFTSAIPEIIFTSAMPQKTYSTMYDHINVNNILKNDRLFNRYFTCLTKRGGCTPEGKLLAAAILDALETSCANCSNEQRKLARQVIQYL

>LstrCSP12 (AGZ04940)
MFKNLLVVCLLVAAVSAKPKPAEKKNTTKYDNIDLDEILNNQRLFDNYYKCLLGAKCTPDGQELKEALPDALATACSKCTEKQRVGTEKVIRHLIEKKPTEYAELEKKYDPQGTYKRKYQAEAIKRGIKV
